# Revision of powdery mildews (*Ascomycota*, *Erysiphaceae*) on *Rosa* in China: unexpected taxonomic complexity with phytopathological implications

**DOI:** 10.3897/imafungus.17.184484

**Published:** 2026-03-04

**Authors:** Dan-Ni Jin, Shuang-Bao Wang, Le-Ping Guan, Xue-Lian Wu, Jing Feng, Li Liu, Michael Bradshaw, Uwe Braun, Jing-Han Yang, Shou-Rong Yu, Yu Li, Shu-Yan Liu

**Affiliations:** 1 College of Plant Protection, Jilin Agricultural University, No. 2888 Xincheng Street, Changchun 130118, Jilin Province, China North Carolina State University Raleigh United States of America; 2 Engineering Research Center of Edible and Medicinal Fungi, Ministry of Education, Jilin Agricultural University, No. 2888 Xincheng Street, Changchun 130118, Jilin Province, China College of Plant Protection, Jilin Agricultural University Changchun China; 3 Key Laboratory of Integrated Pest Management on Crops in Northwestern Oasis, Ministry of Agriculture and Rural Affairs, National Plant Protection Scientific Observation and Experiment Station of Korla, Xinjiang Key Laboratory of Agricultural Biosafety, Institute of Plant Protection, Xinjiang Uygur Autonomous Region Academy of Agricultural Sciences, Urumqi 830091, Xinjiang Uygur Autonomous Region, China Ministry of Education, Jilin Agricultural University Changchun China; 4 Department of Entomology and Plant Pathology, Center for Integrated Fungal Research, North Carolina State University, Raleigh, NC 27606, USA Institute of Plant Protection, Xinjiang Uygur Autonomous Region Academy of Agricultural Sciences Urumqi China; 5 Department for Geobotany and Botanical Garden, Institute of Biology, Martin Luther University, Herbarium, Halle (Saale), 06099, Germany Martin Luther University Halle Germany; 6 Lianyungang Academy of Agricultural Sciences, Lianyungang 222000, Jiangsu Province, China Lianyungang Academy of Agricultural Sciences Lianyungang China

**Keywords:** co-evolution, *
Erysiphe
*, *
Medusosphaera
*, phylogeny, *Podosphaera pannosa*, rose diseases, taxonomic novelties

## Abstract

Powdery mildew is a persistent disease affecting the cultivation of *Rosa*, a genus of substantial horticultural and economic value worldwide. Despite more than a century of study, the true diversity of powdery mildews infecting roses has remained unclear, largely due to the long-standing and overly broad application of the name *Podosphaera pannosa*. To reassess this system, we conducted an extensive investigation of powdery mildew specimens infecting *Rosa*. A total of 112 collections were examined, including recently gathered material from 23 provinces, historical types, representative specimens from the Herbarium Mycologicum Academiae Sinicae (HMAS), China, and a neotype specimen from Germany. Morphological observations combined with phylogenetic analyses (ITS, 28S, and IGS rDNA) resolved several long-standing taxonomic problems and revealed unexpected diversity within the *rose* powdery mildew complex. Molecular data from *Erysiphe rosae* provide the first phylogenetic evidence supporting the synonymy of *Medusosphaera* with *Erysiphe*. *Sphaerotheca rosae*, previously treated as a synonym of *P. pannosa*, is reinstated as a distinct species as *Podosphaera rosae***comb. nov**., and a previously unrecognized lineage is described as *Podosphaera rosae-xanthinae***sp. nov**. In addition, earlier varieties of *E. simulans* are shown to lack diagnostic morphological or genetic characters and are no longer supported. Taken together, these results demonstrate that powdery mildews on *Rosa* represent a complex of five species across two genera, structured by host phylogeny. Clear patterns of host preference and distribution indicate a history of co-evolution and ecological differentiation driven by host availability. This study fundamentally revises our understanding of rose powdery mildews, revealing a level of taxonomic and evolutionary complexity much greater than previously recognized and highlighting *Rosa* as a key host lineage in the diversification of the *Erysiphaceae*.

## Introduction

*Rosa (Rosaceae)* comprises one of the most economically significant horticultural plant genera worldwide. It is native to the Northern Hemisphere, widely distributed in subtropical to cold-temperate regions (Gu and Robertson 2003), and has been extensively cultivated due to its horticultural appeal, culinary uses, and medicinal properties (Božanić Tanjga et al. 2022; Cui et al. 2022; Hegde et al. 2022; Wang et al. 2022; Zhou et al. 2023; Fayaz et al. 2024; Zhang et al. 2024a; Prasad et al. 2025).

China represents a major center of diversity for *Rosa*, with 86 species recorded from the country (Luo et al. 2024). Native Chinese roses, such as *R. chinensis* and *R. odorata*, have provided many important agronomic and ornamental traits for the breeding of modern rose cultivars (Raymond et al. 2018; Yang et al. 2020; Zhou et al. 2020; Zhang et al. 2024b). The cultivation of edible roses in China dates back more than 2,000 years and is concentrated in three major regions: Shandong, Gansu, and Yunnan provinces. Many species have been widely cultivated for edible products, such as rose tea, rose flower cake, rose paste, and rose wine (Jiang 2024). *Rosa* species, such as *R. chinensis*, *R. laevigata*, *R. roxburghii*, and *R. rugosa*, are also used for medicinal purposes in China (Chinese Pharmacopoeia Commission 2020). However, the sustainable production of roses is persistently challenged by powdery mildew. This disease is particularly severe in the cut-flower sector, which constitutes over 80% of China’s floricultural output. Incidences of powdery mildew disease in greenhouses reach 60–90%, with yield losses exceeding 30% (Wang et al. 2018). Furthermore, powdery mildew also inflicts significant damage in the production of edible and medicinal roses, where it causes economic losses of 30–40% (Zuo et al. 2014; Li et al. 2021).

Powdery mildews, a group of obligate biotrophic pathogens within *Erysiphaceae*, encompass approximately 1,000 species across 17 genera and infect around 10,000 angiosperm taxa worldwide (Braun and Cook 2012; Marmolejo et al. 2018; Kiss et al. 2020; Bradshaw et al. 2024, 2025b). China has reported over 300 species of powdery mildew fungi (Zheng and Yu 1987; Tang et al. 2017; Tang et al. 2018; Qiu et al. 2019; Jin et al. 2021; Feng et al. 2022, 2025a; Liu et al. 2022; Zhang et al. 2025), among which four species and one variety across three genera have been reported to infect roses, including *Medusosphaera rosae* (now known as *Erysiphe rosae*), *Sphaerotheca pannosa* (now known as *Podosphaera pannosa*), *S. rosae* (currently considered a synonym of *P. pannosa*), and *Uncinuliella simulans var. rosae-rubi* (now known as *Erysiphe simulans var. rosae-rubi*) (Zheng and Yu 1987; Braun and Cook 2012). This assemblage encompassed nearly all powdery mildew species known globally on *Rosa*. However, the name *Podosphaera pannosa* has become a catch-all designation for powdery mildew on roses during the past century. The traditional focus on *P. pannosa* has contributed to persistent taxonomic confusion and limited recognition of diversity within rose powdery mildews. The genus *Medusosphaera*, with *M. rosae* as its type species (Golovin and Gamalitskaya 1962), was previously reduced to synonymy with *Erysiphe*, as the differences in the morphology of the appendages were regarded as insufficient to maintain it as an independent genus (Braun and Takamatsu 2000). However, so far, this treatment has not been supported by molecular data. In addition, critical species-level ambiguities persist. For example, the recognition of two *Sphaerotheca* species associated with rose powdery mildew prevailed in previous taxonomic concepts (Salmon 1900; Jaczewski 1927; Blumer 1933, 1967). One is the well-known, previously mentioned species, *Podosphaera pannosa*, whereas the other species, *Sphaerotheca rosae*, is a combination introduced by Zhao (1981) based on Jaczewski’s (1927) *S. macularis f. rosae*. Zheng and Yu (1987) accepted Zhao’s treatment, whereas most subsequent authors considered this name a synonym of *P. pannosa* (Braun 1987, 1995; Shin 2000; Liu 2010; Braun and Cook 2012). The relationship between the commonly reported *P. pannosa* and the previously proposed *S. rosae* has remained unclear until recently due to the lack of detailed morphological and molecular comparison of type specimens. Additionally, Bradshaw et al. (2023) regarded the varieties of *E. simulans* as dubious due to indistinct morphological boundaries and the lack of genetic differences between *E. simulans var. rosae-rubi* and *E. simulansvar. tandae* and emphasized the need for analyses of additional specimens and loci for this species. Consequently, the narrow emphasis on *P. pannosa* has resulted in an incomplete taxonomic framework for rose powdery mildews, constraining analyses of host range, geographic distribution, and genetic diversity.

These gaps hinder the evaluation of host-associated diversification of powdery mildew fungi on *Rosa*. As obligate fungi, powdery mildews typically share a close relationship with their host plants. For instance, co-evolutionary relationships have been reported in both the *Golovinomyces*–*Asteraceae* and *Podosphaera*–*Rosaceae* systems (Matsuda and Takamatsu 2003; Takamatsu et al. 2010). However, it remains unclear whether and how *Rosa*, a genus with high diversity at the species level, affects the species diversity and distribution patterns of powdery mildews. This lack of knowledge consequently impedes the development of targeted control measures against powdery mildew on *Rosa*.

Therefore, we sought to establish a comprehensive taxonomic framework for powdery mildews on *Rosa*. Using nationwide sampling in China and an integrative approach combining morphology with phylogenetic analyses of the ITS region (including 5.8S rDNA), partial 28S rDNA (D1–D2 domains), and the intergenic spacer (IGS), we (1) resolved longstanding taxonomic confusion and clarified species diversity of *rose* powdery mildews in China and globally; (2) characterized host associations and distribution patterns; and (3) evaluated relationships between these fungi and their *Rosa* hosts.

## Materials and methods

### Specimen collection

A comprehensive investigation was conducted across 30 provinces in China. A total of 97 *rose* powdery mildew specimens from 23 provinces were obtained and deposited in the Herbarium of Mycology of Jilin Agricultural University (HMJAU). An additional 15 specimens were analyzed, of which 14 were type or representative specimens of *Medusosphaera rosae* (5), *Uncinuliella simulans var. simulans* (1), *Un. simulans var. rosae-rubi* (1), *Sphaerotheca rosae* (4), and *Podosphaera pannosa* (3), which were borrowed from the Herbarium Mycologicum Academiae Sinicae (HMAS), and one German specimen from the Martin-Luther-Universität Herbarium (HAL), which is designated as the neotype for *P. pannosa*. In total, 112 dried herbarium specimens were examined in this study. Detailed information about these specimens is shown in Suppl. material 1.

### Morphological examination

The sample preparation procedure followed Feng et al. (2025b). For anamorph examinations of dried specimens, the parts of the plants with the most abundant powdery mildew colonies were cut off using a sterile surgical blade and immersed in lactic acid, with the powder layer facing downward on the slide. For teleomorph examinations of dried specimens, chasmothecia were placed onto a drop of lactic acid on the slide. The slide was then gently heated, and the plant tissue was removed before microscopic examination with a phase-contrast light microscope (ZEISS Scope A1, Germany).

### DNA extraction and PCR amplification

Genomic DNA was extracted from conidia, mycelia, or chasmothecia using the Chelex-100 method (Walsh et al. 1991; Hirata and Takamatsu 1996). The ITS, 28S, and IGS rDNA regions were generated using polymerase chain reaction (PCR) for molecular analyses. Primers used for amplification are listed in Table 1. The amplification reactions were performed in a total volume of 25 μL reaction mixture, including 2 μL template DNA, 12.5 μL Premix Taq [TaKaRa Taq 1.25 U/25 μL, 2× dNTP Mixture (0.4 mM each), 2× Taq Buffer (3 mM Mg^2+^)] (TaKaRa, Tokyo, Japan), 1 μL of each primer (10 μM), and 8.5 μL ddH_2_O. The PCR reactions were conducted under the following thermal cycling conditions: an initial predenaturation step of 5 min at 95 °C, followed by 35 cycles of 30 s at 94 °C for denaturation, 30 s at 52–58 °C for annealing (temperatures for different primer pairs are listed in Table 1), and 1 min at 72 °C for extension, and a final extension step of 10 min at 72 °C. The PCR products were subjected to electrophoresis in a 1.2% agarose gel in 0.5× TBE buffer. The amplicons were sent to Sangon Biotech (Shanghai, China) for direct sequencing of both strands using the same primers as for the PCR. The assembled sequences were deposited in the National Center for Biotechnology Information (NCBI) GenBank nucleotide database, and the accession numbers are listed in Suppl. material 1.

**Table 1.** Primer pairs and annealing temperatures used in this study.

Locus	Primer pair (forward/reverse)	Sequence (5’–3’)	Annealing temperature (°C)	Reference
ITS	ITS5/ITS4	GGAAGTAAAAGTCGTAACAAGG/ TCCTCCGCTTATTGATATGC	56	White et al. 1990
	PM1/PM2	TCGGACTGGCCYAGGGAGA/ TCACTCGCCGTTACTGAGGT	55	Cunnington et al. 2003
	PM10/PM11	GGCCGGAAAGTTGTCCAAAC/ TACCGCTTCACTCGCCGTTA	56	Bradshaw and Tobin 2020
28S	PM3/TW14	GKGCTYTMCGCGTAGT/ GCTATCCTGAGGGAAACTTC	52	Mori et al. 2000
	LSU1/NLP2	ACCCGCTGAACTTAAGCATA/ GGTCCCAACAGCTATGCTCT	52	Scholin et al. 1994; Mori et al. 2000
IGS	IGS-12a/NS1R	AGTCTGTGGATTAGTGGCCG/ GAGACAAGCATATGACTAC	58	Carbone and Kohn 1999

### Phylogenetic analyses

Sequences of ITS, 28S, and IGS newly generated in this study, along with related sequences from GenBank, were automatically aligned using MUSCLE in MEGA X separately (Kumar et al. 2018) and then manually adjusted. Sequence alignments from different fragments were concatenated using PhyloSuite v1.2.3 (Zhang et al. 2019; Xiang et al. 2023) to construct a concatenated matrix.

Three datasets were then constructed: a 28S rDNA dataset of *Erysiphaceae* for clarifying the phylogenetic position of *Medusosphaera* in *Erysiphaceae*, with *Byssoascus striatosporus* as the outgroup; an ITS+28S+IGS dataset of the genus *Erysiphe* for clarifying the phylogenetic position of *Erysiphe rosae* and exploring whether there are genetic differences among varieties of *E. simulans*, with both *Bulbomicroidium bauhiniicola* and *Brasiliomyces malachrae* as outgroups; and an ITS+28S+IGS dataset of *Podosphaera* sect. *Sphaerotheca* with all credible sequences of *P. pannosa* from GenBank for clarifying the phylogenetic relationship of *P. pannosa* and *Sphaerotheca rosae*, with *Cystotheca lanestris* as the outgroup. The accession numbers for the sequences from GenBank are indicated on the corresponding phylogenetic trees. Alignments were submitted as Suppl. materials 2–4.

Phylogenetic analyses were conducted using the maximum parsimony (MP), maximum likelihood (ML), and Bayesian inference (BI) methods. MP analyses were conducted in PAUP* 4.0 (Swofford 2002), identifying optimal trees through a heuristic search with tree-bisection-reconnection (TBR) branch swapping and 1,000 random sequence additions to thoroughly explore tree space. All gaps were treated as missing data, and sites were treated as unordered and unweighted. The ML analyses were performed in raxmlGUI v2.0-beta (Edler et al. 2021) using the GTRGAMMA evolutionary model. Bootstrap supports and trees were obtained by running a rapid bootstrap analysis of 1,000 replicates. For BI analyses, the best-fit substitution models were estimated using MrModeltest v2.3 (Nylander 2004) based on the implementation of the Akaike information criterion (AIC). Four Markov chain Monte Carlo (MCMC) chains were run using MrBayes v3.2.7 (Ronquist and Huelsenbeck 2003). The analysis ran for 3 M generations, and trees were sampled every 100 generations. The first 25% of trees were discarded as burn-in, and a majority-rule consensus tree of all remaining trees was calculated to determine the posterior probabilities for individual branches. The runs were stopped when the standard deviation of split frequencies reached below 0.01. All methods produced congruent topologies, with horizontal branch lengths in the selected MP tree reflecting inferred substitutions. The best-scoring tree from the MP analysis was further edited using Adobe Illustrator 2024. Bootstrap values from BI and ML analyses were superimposed on the MP framework.

### Host preference

To elucidate the association between powdery mildews and their *Rosa* hosts, the relative frequency (RF) was calculated to present the host frequency of rose powdery mildews. For each rose powdery mildew species, the respective distribution among different *Rosa* sections and species was analyzed. The frequency of individual powdery mildew species on a particular host section or species was calculated as RF (%) = (f / N) × 100. Here, f represents the number of specimens of a powdery mildew species from a specific *Rosa* section or species, and N is the total number of specimens identified as the powdery mildew species concerned. This study involved 13 *Rosa* species across four sections: sect. *Chinenses* (C), sect. *Pimpinellifoliae* (Pi), sect. *Rosa* (R), and sect. *Synstylae* (S). Because sections *Chinenses* and *Synstylae* are not reciprocally monophyletic and form a nested (paraphyletic) relationship (Cheng et al. 2025), we treated them as a single group (*Chinenses* + *Synstylae*, CS) in this analysis.

### Distribution patterns and co-distribution analysis with primary hosts

The accurate locations of 110 Chinese specimens (Suppl. material 1) were used in the distribution analysis. The distribution maps were drawn using ArcGIS 10.8 (Esri, Redlands, CA, USA). To clarify the distribution patterns of rose powdery mildews in China, the coordinates of all 110 specimens were inserted into a map as point features, with different species labeled in different colors. To explore whether there are co-distributions of rose powdery mildews and their hosts, separate maps were generated for each powdery mildew species, overlaid with the distribution range of its primary host. The distribution areas in China of the corresponding primary hosts are based on the *Rosa* monograph of Luo et al. (2024) and are marked in pink on the maps.

## Results

### Phylogenetic analyses

A total of 292 sequences were obtained in this study, comprising 102 ITS, 101 28S, and 89 IGS sequences (Suppl. material 1). Four phylogenetic trees were constructed based on these newly generated sequences and those retrieved from GenBank.

**Figure 1.**
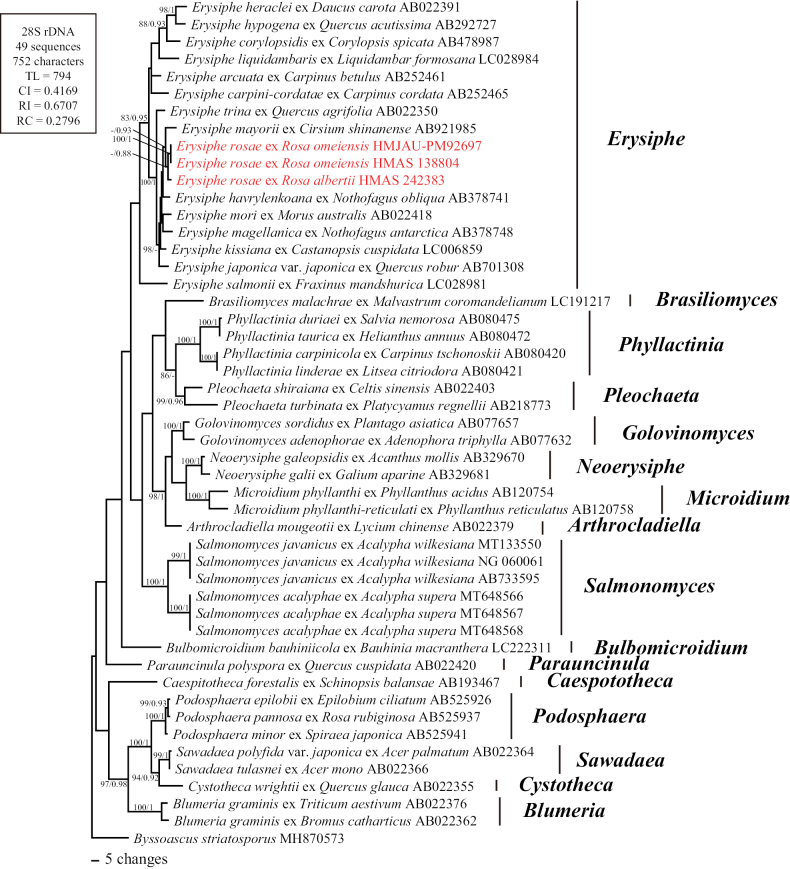
Phylogenetic analysis of *Erysiphaceae* based on 28S rDNA sequences. Bootstrap values larger than 80% for the maximum likelihood (ML) analyses conducted are displayed, followed by posterior probabilities ≥80. The newly generated sequences in this study are highlighted in red. *Byssoascus striatosporus* is used as the outgroup taxon.

The 28S sequence alignment matrix of *Erysiphaceae* totaled 752 characters and consisted of 49 sequences, including three newly generated and 45 retrieved from GenBank. The maximum likelihood (ML) bootstrap supports greater than 80%, and Bayesian posterior probabilities (BPP) over 0.8 are shown in Fig. 1. In this phylogenetic tree, sequences of *Erysiphe rosae* cluster within the *Erysiphe* clade, confirming the previously proposed synonymy of *Medusosphaera* and *Erysiphe*.

**Figure 2.**
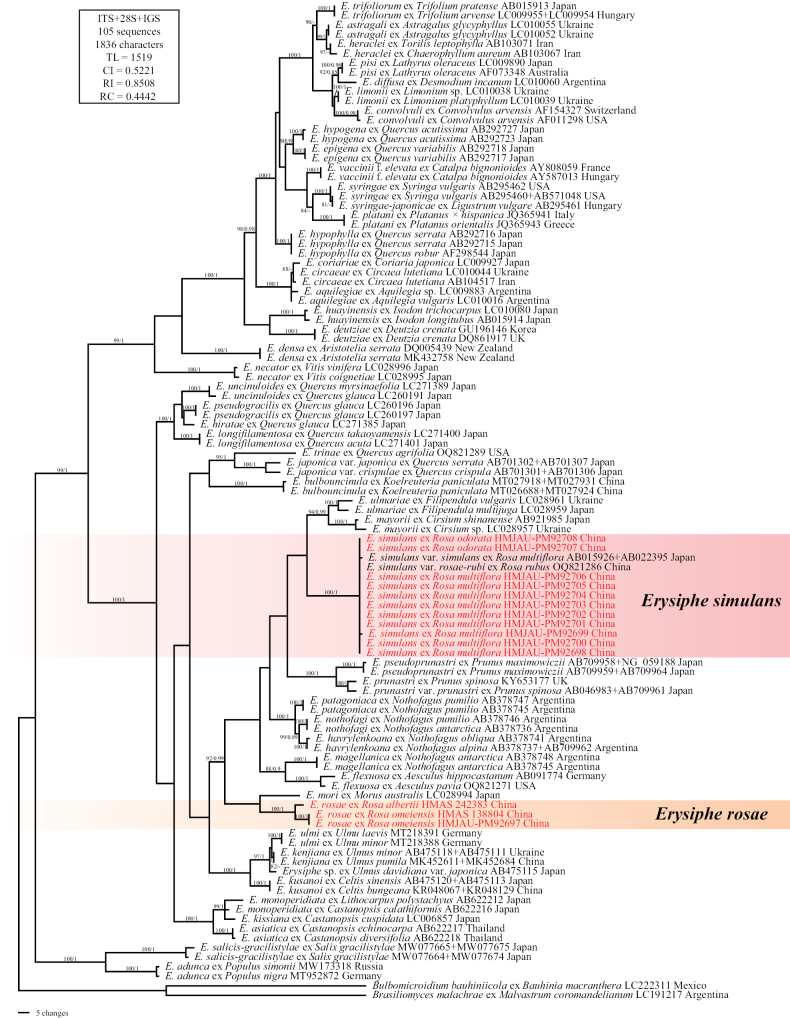
Phylogenetic analysis of related *Erysiphe* species based on ITS + 28S + IGS sequences. Bootstrap values larger than 80% for the maximum likelihood (ML) analyses conducted are displayed, followed by posterior probabilities ≥80. The newly generated sequences in this study are highlighted in red. *Bulbomicroidium bauhiniicola* and *Brasiliomyces malachrae* are used as outgroup taxa.

The ITS+28S+IGS sequence alignment matrix of *Erysiphe* comprised 1,836 characters and consisted of 105 sequences, including 14 newly generated and 91 retrieved from GenBank. The maximum likelihood (ML) bootstrap supports greater than 80%, and Bayesian posterior probabilities (BPP) over 0.8 are shown in Fig. 2. Sequences of *E. simulans* from this study and GenBank form a highly supported monophyletic group. Sequences of *E. rosae* vary across different hosts, which may indicate cryptic speciation (Fig. 2).

**Figure 3.**
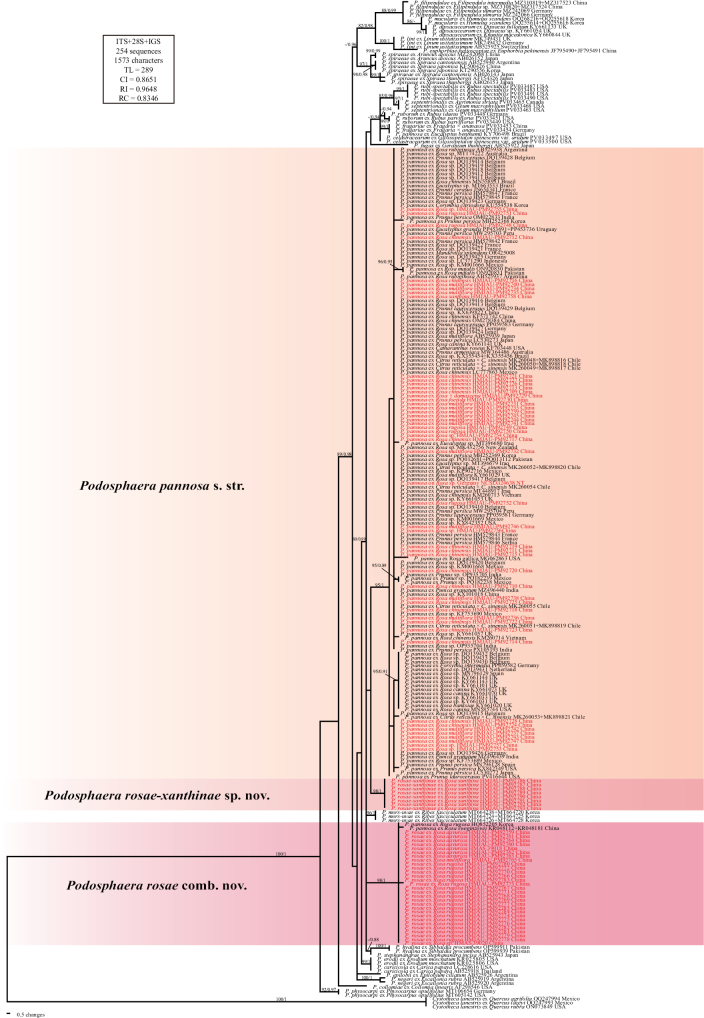
Phylogenetic analysis of related *Podosphaera* sect. *Sphaerotheca* species based on ITS + 28S + IGS sequences. Bootstrap values larger than 80% for the maximum likelihood (ML) analyses conducted are displayed, followed by posterior probabilities ≥80. The newly generated sequences in this study are highlighted in red. *Cystotheca lanestris* is used as the outgroup taxon.

The ITS+28S+IGS sequence alignment matrix of *Podosphaera* sect. *Sphaerotheca* totaled 1,573 characters and consisted of 254 sequences, including 88 newly generated and 166 retrieved from GenBank. The maximum likelihood (ML) bootstrap supports greater than 80%, and Bayesian posterior probabilities (BPP) over 0.8 are shown in Fig. 3. The phylogenetic tree showed that sequences of *Podosphaera* on *Rosa* hosts cluster into three distinct clades. The ex-neotype sequences of *P. pannosa* (PV581762 + PV584310) and ex-type sequences of *Sphaerotheca rosae* (PX239400 + PX239279) fall into separate clades, providing definitive molecular evidence that *S. rosae* is a species distinct from *P. pannosa*. Moreover, sequences retrieved from powdery mildew on *Rosa xanthina* form a well-supported clade separate from *P. pannosa*, suggesting the presence of a novel *Podosphaera* species on *Rosa*.

### Taxonomy

Integrated morphological and phylogenetic analyses led to the following taxonomic conclusions: 1) confirmation of *Medusosphaera* as a synonym of *Erysiphe*; 2) confirmation that the previously described varieties of *E. simulans* do not have taxonomic value and can be dropped; 3) clarification that *Podosphaera rosae* comb. nov. is an independent species distinct from *P. pannosa* s. str.; and 4) description of a new species, *P. rosae-xanthinae* sp. nov. Detailed descriptions and notes are provided below.

#### Erysiphe rosae

Taxon classificationFungiHelotialesErysiphaceae

(Golovin & Gamalitsk.) U. Braun & S. Takam., Schlechtendalia 4: 4, 2000

CECF8F03-0AD5-568B-AE7E-9110C260BDB5

Fig. 4

≡ Medusosphaera rosae Golovin & Gamalitsk., Bot. Mater. Otd. Sporov. Rast. Bot. Inst. Komarova Akad. Nauk S.S.S.R. 15: 92, 1962.

##### Type.

***Holotype***: Kyrgyzstan • Tjan-Shan, Kavak-Tau, Tabylgyty, on *Rosa albertii*, 14 Sep. 1959, Gamalitskaya (LE 193792). Reference collection: China, Xinjiang, Urumqi, on *Rosa albertii*, 3 Aug. 2009, Z.Y. Zhao & B. Xu (HMAS 242383). Reference sequences: PX506005 (ITS), PX506007 (28S), PX570626 (IGS).

**Figure 4.**
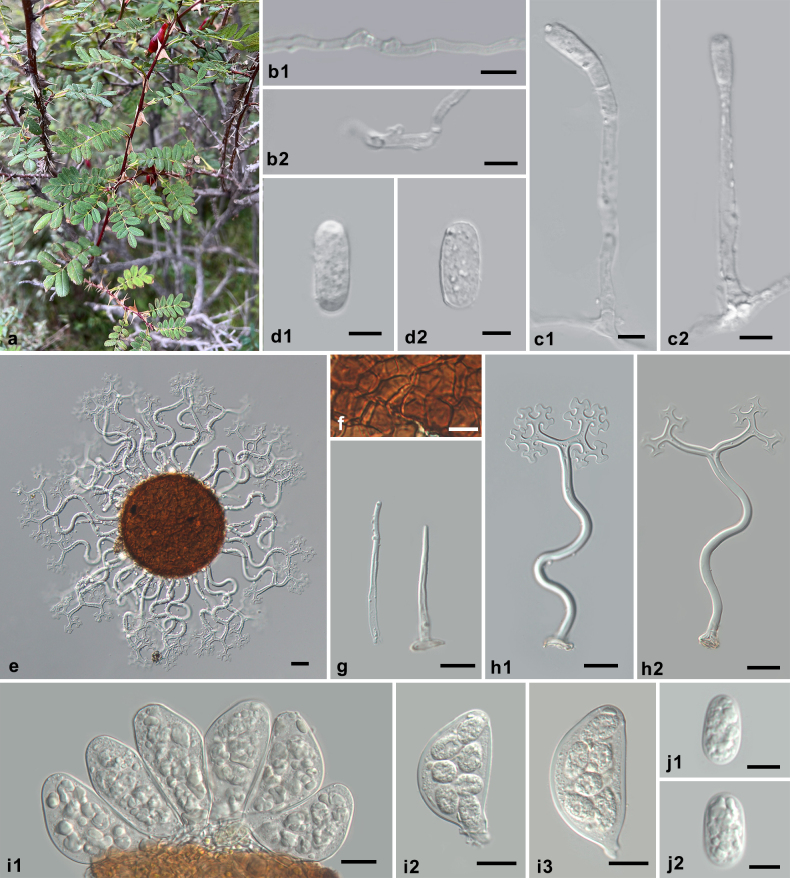
Symptoms and morphology of *Erysiphe rosae* on *Rosa omeiensis* (HMJAU-PM92697). **a** Symptoms. **b1, b2** Hyphal appressoria. **c1, c2** Conidiophores with conidia formed singly. **d1, d2** Conidia. **e** Chasmothecium. **f** Peridium cells. **g** Short appendages. **h1, h2** Long appendages. **i1–i3** Asci. **j1, j2** Ascospores. Scale bars: 10 μm (**b1, d2, f, j1, j2**); 20 μm (**e, g–i3**).

##### Specimens examined.

• HMJAU-PM92697, HMAS 36517, HMAS 36518, HMAS 44287, and HMAS 138804. Detailed specimen information is presented in Suppl. material 1.

##### Morphological description.

Braun and Cook (2012: 501–502).

##### Host range and distribution.

on *Rosa* (albertii, *amblyotis*, davurica, omeiensis, *webbiana*), *Rosaceae*; Asia (China, India, Kyrgyzstan, Russia, Far East).

##### Notes.

*Erysiphe rosae* is characterized by sinuous long appendages and bristle-like short appendages. This taxon has been controversially treated at the generic level. It was first introduced as the type species of the genus *Medusosphaera* and later transferred to *Erysiphe* because the morphology of the chasmothecial appendages was no longer recognized as suitable for differentiation at the generic level (Golovin and Gamalitskaya 1962; Braun and Takamatsu 2000). The correct allocation of *M. rosae* to *Erysiphe* was confirmed in the present study based on lobed hyphal appressoria and conidiophores with singly formed conidia (Fig. 4), as well as sequences successfully retrieved from specimens of this species, which form a highly supported species clade within *Erysiphe* (Fig. 1). It should be noted that the sequences of the three examined specimens clustered together according to their host plants, which indicates previously unrecognized diversity within *E. rosae*, possibly suggesting cryptic formae, a taxonomic unit recently reapplied in *Erysiphaceae* (Bradshaw et al. 2025c; Feng et al. 2025a). However, due to the limited number of specimens available, it was impossible to obtain additional molecular data or to observe potential morphological differences. Therefore, following the species concept proposed by Bradshaw et al. (2022), we provisionally treated these specimens as *E. rosae* s. lat. Since the holotype of this species from Kyrgyzstan has not yet been sequenced, the Chinese specimen HMAS 242383 on the type host *Rosa albertii* is selected as the reference collection with reference sequences for the interim.

#### Erysiphe simulans

Taxon classificationFungiHelotialesErysiphaceae

(E.S. Salmon) U. Braun & S. Takam., Schlechtendalia 4: 23, 2000

A58C0F16-B9A6-5965-8855-B877253C0723

Figs 5, 6

≡ Uncinula simulans E.S. Salmon, Ann. Mycol. 6: 2, 1908.
≡ Uncinuliella simulans (E.S. Salmon) R.Y. Zheng & G.Q. Chen, Acta Microbiol. Sin. 19(3): 286, 1979.
= Uncinuliella simulans var. *rosae-rubi* R.Y. Zheng & G.Q. Chen, Acta Microbiol. Sin. 19(3): 288, 1979.
≡ Erysiphe simulans var. *rosae-rubi* (R.Y. Zheng & G.Q. Chen) U. Braun & S. Takam., Schlechtendalia 4: 23, 2000.
= Uncinuliella simulans var. tandae U. Braun, Mycotaxon 22(1): 92, 1985.
≡ Erysiphe simulans var. tandae (U. Braun) U. Braun & S. Takam., Schlechtendalia 4: 24, 2000.

##### Type.

***Lectotype*** (designated by Braun 1987): Japan • Morioka, on *Rosa multiflora*, 21 Oct. 1906, Sawada (TNS-F-214613). Reference sequences (designated by Bradshaw et al. 2023): AB015926 (ITS), AB022395 (28S) [in Takamatsu et al. (1999) and Mori et al. (2000)].

**Figure 5.**
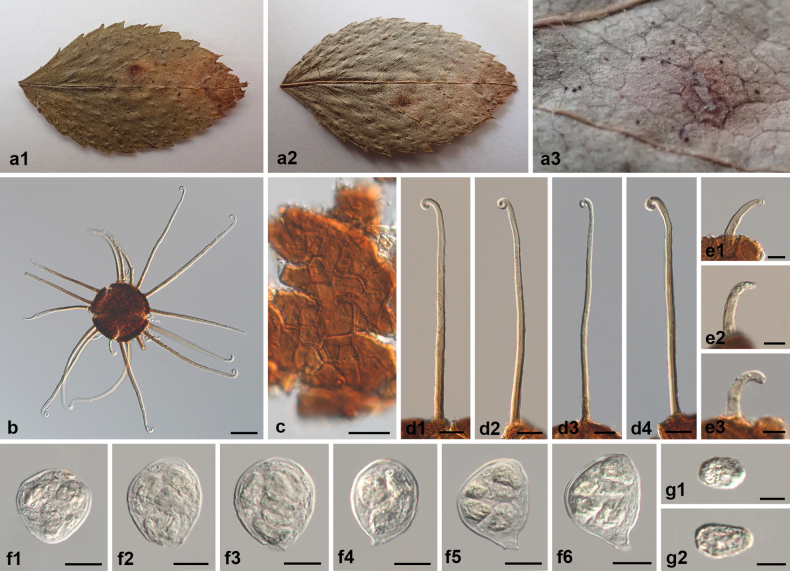
Symptoms and morphology of *Erysiphe simulans* on the type host, *Rosa multiflora* (HMAS 13631). **a1–a3** Symptoms. **b** Chasmothecium. **c** Peridium cells. **d1–d4** Long appendages. **e1–e3** Short appendages. **f1–f6** Asci. **g1, g2** Ascospores. Scale bars: 50 μm (**b**); 20 μm (**c–d4, f1–f6**); 10 μm (**e1–e3, g1, g2**).

**Figure 6.**
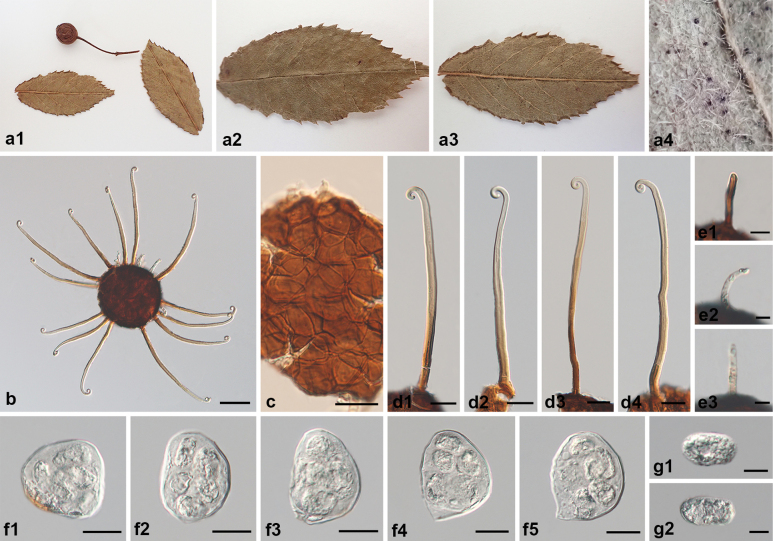
Symptoms and morphology of *Erysiphe simulans* “var. *rosae-rubi*” on *Rosa rubus* (HMAS 11418). **a1–a4** Symptoms. **b** Chasmothecium. **c** Peridium cells. **d1–d4** Long appendages. **e1–e3** Short appendages. **f1–f5** Asci. **g1, g2** Ascospores. Scale bars: 50 μm (**b**); 20 μm (**c–d4, f1–f5**); 10 μm (**e1–e3, g1, g2**).

##### Specimens examined.

• A total of 13 specimens were examined, including HMJAU-PM92698 to HMJAU-PM92708, and two specimens from the Herbarium Mycologicum Academiae Sinicae, HMAS 11418 and HMAS 13631. Detailed specimen information is presented in Suppl. material 1.

##### Morphological description.

Braun and Cook (2012: 586).

##### Host range and distribution.

on *Rosa* (multiflora, odorata, rubus), *Rosaceae*; Asia (China, Japan, Korea).

##### Notes.

Bradshaw et al. (2023) found that the sequences of E. simulans var. rosae-rubi and *E. simulans var. tandae* grouped together with high bootstrap values. The authors reported that the morphological differences were only gradual or had overlapping quantitative features and, as such, concluded that varieties under *E. simulans* should be discarded. The morphology of a reference specimen of *E. simulans* from Japan (HMAS 13631) and the holotype of *E. simulans* var. *rosae-rubi* from China (HMAS 11418) were re-examined in this study (Figs 5, 6). We confirmed that the two are highly similar in all characteristics. The differences in the number of appendages described by Zheng and Chen (1979) are also indistinct and overlap with each other. Although we failed to amplify sequences from those two specimens, other sequences of *E. simulans* were obtained from eleven newly collected specimens. The present phylogenetic analysis showed that there are minimal genetic differences within this species. Therefore, it is reasonable and justified to discard these varieties of *E. simulans*.

#### Podosphaera pannosa

Taxon classificationFungiHelotialesErysiphaceae

(Wallr.) de Bary, Abh. Senkenb. Naturf. Ges. 7: 408, 1870. s. str. [emend]

17EF615C-5662-5F30-A2A4-2B9457C570C7

Fig. 7

≡ *Alphitomorpha pannosa* Wallr., Verh. Ges. Naturf. Freunde Berlin 1: 43, 1819.
≡ Erysiphe pannosa (Wallr.) Link (as “Erysibe”), in Willdenow, Sp. pl., Edn 4, 6(1): 104, 1824, nom. sanct. (Fr., Syst mycol. 3(1): 236, 1829).
≡ Sphaerotheca pannosa (Wallr.) Lév., Ann. Sci. Nat., Bot., 3 Sér., 15: 138, 1851.

##### Type.

***Neotype*** (designated here, MBT10030088): Germany • Lower Saxony, Hannover-Ahlem, experimental section “cemetery planting,” on *Rosa* sp. cult. (hybrid), 27 Sep. 2018, P. Houska (NCSLG24638). ***Isoneotype***: HAL 3600 F. Ex-neotype sequences: PV581762 (ITS+28S), PV584310 (IGS), PV584347 (TUB).

**Figure 7.**
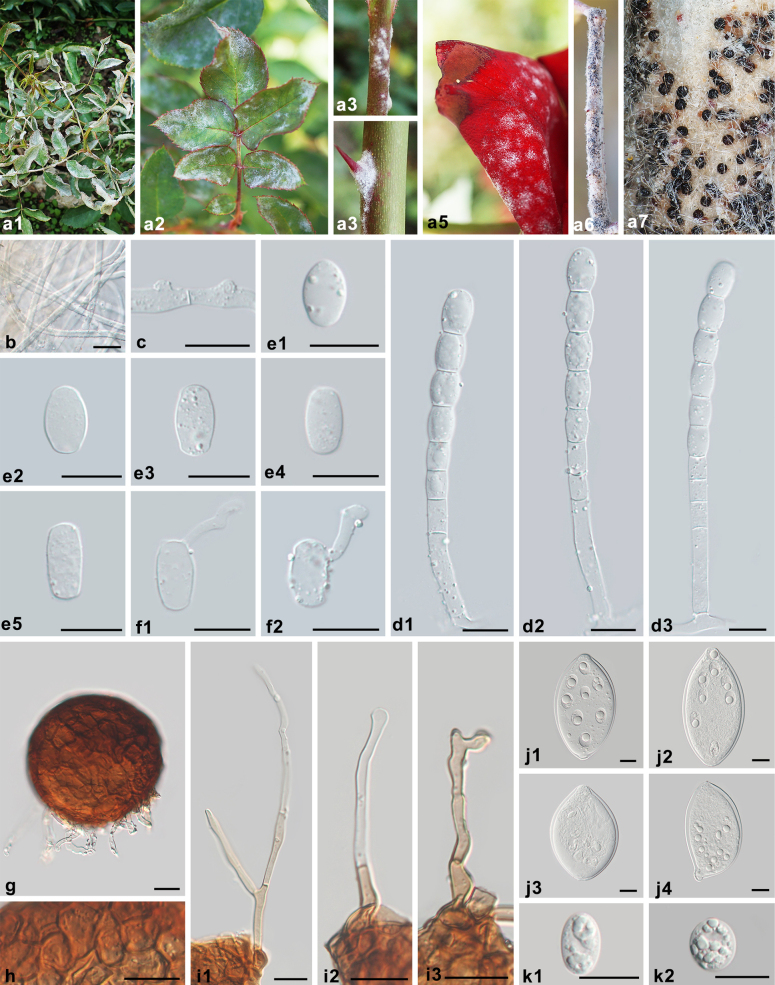
Symptoms and morphology of *Podosphaera pannosa* on *Rosa chinensis* (HMJAU-PM92727). **a1–a7** Symptoms. **b** Secondary hyphae. **c** Hyphal appressoria. **d1–d3** Conidiophores with catenescent conidia. **e1–e5** Conidia. **f1, f2** Germ tubes. **g** Chasmothecium. **h** Peridium cells. **i1–i3** Appendages. **j1–j4** Asci. **k1, k2** Ascospores. Scale bars: 20 μm (**b–k2**).

##### Description.

Mycelium on leaves, amphigenous, also on stems and petals, persistent, infected shoots often curled and distorted, forming irregular patches or effuse; primary hyphae hyaline, thin-walled, straight or sometimes flexuous, (2.5–)3–5.5 μm wide; secondary hyphae hyaline, thick-walled, 4–6 μm wide, colonies on twigs only developed in the later stages of the disease; hyphal appressoria indistinct to nipple-shaped, solitary or side by side; conidiophores arising from the upper surface of mother cells, erect, (52–)60–97(–99) × (6.5–)7.5–9.5 μm, foot cells straight, cylindrical, 33–59 × 7–9.5 μm, followed by (1–)2–3 shorter cells, forming catenescent conidia; conidia variable, primary conidia obovoid, secondary conidia doliiform to cylindrical, 20–26 × 11.5–15 μm, length/width ratio 1.5–2.1; germ tubes almost terminal. Chasmothecia gregarious, subglobose to globose, immersed in the secondary mycelial patches, mostly on stems, but also produced on leaves when the disease is severe, (74–)88–113 μm diam.; peridium cells round to irregularly polygonal, 4.0–16 μm diam.; appendages few, in the lower half of chasmothecia, mycelioid, short, usually shorter than chasmothecial diam., rarely up to 1.5 times diam., occasionally branching, brownish at the basal part or brown throughout, septate, thin-walled, smooth, coarse; ascus ovoid to fusiform, colorless, thick-walled, sessile or almost sessile, 87–140 × 63–84 μm, length/width ratio 1.2–1.9, terminal oculus obvious, large, 13.5–21 μm, 8-spored; ascospores ellipsoid to round, colorless, (16.5–)18–25 × 12.5–17.5 μm, length/width ratio 1.1–1.9.

##### Additional specimens examined.

• A total of 51 specimens were examined, including 50 specimens with voucher numbers HMJAU-PM92709 to HMJAU-PM92758 and one specimen of HMAS 38723. Detailed specimen information is presented in Suppl. material 1.

##### Host range and distribution.

(phylogenetically confirmed hosts and distribution). *Rosa* (*canina*, chinensis, × *damascena*, *foetida*, *gallica*, *laxa*, *majalis*, multiflora, *albertiirubiginosa*, rugosa, xanthina), *Prunus* (*armeniaca*, *cerasus*, *laurocerasus*, *persica*, *granatum*), *Rosaceae*; *Catharanthus roseus*, *Apocynaceae*; *Citrus reticulata* × *C. sinensis*, *Rutaceae*; *Corymbia citriodora*, *Eucalyptus* (*benthamii*, *grandis*, sp.), *Myrtaceae*; and *Forsythia × intermedia*, *Oleaceae*. Asia (China, India, Iraq, Israel, Japan, Pakistan, South Korea, Vietnam), Europe (Belgium, France, Germany, Netherlands, Serbia, Spain, United Kingdom), North America (Mexico, United States), Oceania (Australia, New Zealand), and South America (Argentina, Brazil, Chile, Peru, Uruguay).

##### Notes.

*Podosphaera pannosa* is the most widely distributed species on *Rosa* spp. *Alphitomorpha pannosa*, the basionym, was described from Germany, but type material is not preserved (Braun and Cook 2012). Therefore, a German specimen is designated as the neotype with ex-neotype ITS, 28S, IGS, and TUB sequences. Based on the current morphological and phylogenetic results, it is clear that *P. rosae* is genetically distinguishable from *P. pannosa*. The records of *P. pannosa* on *R. rugosa* (HMAS 01630, 14132) in Zheng and Yu (1987) from China should probably be revised to *P. rosae* after re-examining the specimens. The description and illustration of *P. pannosa* in Shin (2000) from Korea are also more in line with *P. rosae* and in need of revision. There are numerous morphological descriptions of *P. pannosa* that appear to be composed of characteristics of both *P. pannosa* and *P. rosae* (Braun 1987, 1995; Liu 2010; Braun and Cook 2012).

The confusions and misidentifications were documented as early as Salmon’s monograph (1900). He postulated that the primary reason for this confusion was the erroneous assumption that the species of fungus that occurs on a certain host plant in one part of the world will be the same as that growing on the same host in other parts of the world. As such, many powdery mildews on *Rosa* spp. were identified as *P. pannosa*, and the other possible species were ignored. In addition, the chasmothecia of *P. pannosa* are rarely formed (Salmon 1900; Blumer 1933; Homma 1937; Junell 1967; Nomura 1997), so the fact that chasmothecia of *P. rosae* appear to be more common than those of *P. pannosa* may be another reason for the confusion. To our knowledge, *P. rosae* often forms chasmothecia in China in late autumn when the temperature decreases. Chasmothecia of *P. pannosa* are rarely formed, even when chasmothecia of *P. rosae* are formed at the same time and place. The chasmothecia of *P. pannosa* can be found in winter or even the following spring, and they are primarily formed on stems, rarely on leaves, with abundant secondary mycelium. The more frequently produced chasmothecia of *P. rosae* are undoubtedly the reason that they have often erroneously been assigned to *P. pannosa*, at least before these two species were separated. Consequently, on account of the persistent taxonomic confusion between these two species, the distribution and host range require further clarification. In this study, host range and distribution are listed based on the results of the phylogenetic analysis (see Fig. 3).

#### Podosphaera rosae

Taxon classificationFungiHelotialesErysiphaceae

(Jacz.) D.-N. Jin & S.-Y. Liu comb. nov.

939B626C-C809-5AA2-B5B5-8DC411D12293

861536

Fig. 8

≡ Sphaerotheca rosae (Jacz.) Z. -Y. Zhao, Acta Microbiol. Sin. 4: 439, 1981.

##### Basionym.

*Sphaerotheca macularis f. rosae* Jacz., Karmannyi opredelitel’ gribov II. Muchnistorosyanye griby: 76. 1927.

**Figure 8.**
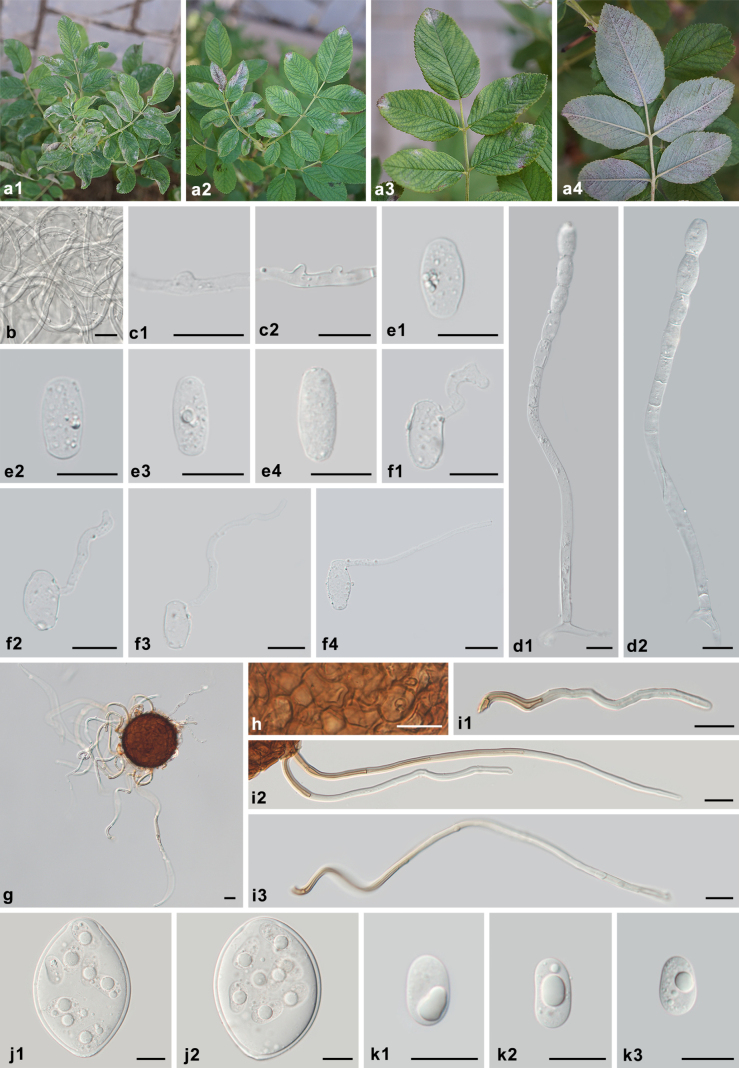
Symptoms and morphology of *Podosphaera rosae* on *Rosa rugosa* (HMJAU-PM92763). **a1–a4** Symptoms. **b** Secondary hyphae. **c1, c2** Hyphal appressoria. **d1, d2** Conidiophores with catenescent conidia. **e1–e4** Conidia. **f1–f4** Germ tubes. **g** Chasmothecium. **h** Peridium cells. **i1–i3** Appendages. **j1, j2** Asci. **k1–k3** Ascospores. Scale bars: 20 μm (**b–k3**).

##### Type.

***Lectotype*** (designated here, MBT10029826), • original illustration in Jaczewski (1927: 77, fig. 19). ***Epitype*** (designated here, MBT10030086): China • Shaanxi, Yulin, on *Rosa davurica*, 18 Oct. 2023, L. Liu & Z.-Y. Zhang (HMJAU-PM92763). Ex-epitype sequences: PX239405 (ITS), PX239284 (28S), PX239896 (IGS).

Mycelium on stems and both sides of leaves, more abundant on the lower surfaces of the leaves, infected shoots often curled and distorted, forming irregular patches or effuse, persistent; primary hyphae hyaline, thin-walled, 3.0–4.5 μm wide, secondary hyphae hyaline, thick-walled, 3.5–6 μm wide, sparsely branched, often intertwined with each other on the lower surface of the leaves; hyphal appressoria indistinct to nipple-shaped, solitary or side by side; conidiophores arising from the upper surface of mother cells, erect, very long, 65–240 (–342) × 7–10.5 μm, foot cells straight, cylindrical, long, 31–195(–302) × 7–10.5 μm, followed by 2–3 shorter cells, forming catenescent conidia; conidia doliiform to ellipsoid, 22–29(–31) × 11.5–15.5(–16.5) μm, length/width ratio 1.5–2.3; germ tubes almost terminal or terminal, short or long. Chasmothecia scattered, subglobose to globose, amphigenous, mostly on the lower surface of leaves, (74–)77–101(–106) μm diam.; peridium cells round to irregularly polygonal, 6.5–17.5 μm diam.; appendages mycelioid, some of them slightly spirally twisted at the base, arising from the equator of the chasmothecia, sometimes curved, wall thick and smooth, light brown below, paler towards the apex, numerous, various in length, (59–)80–405(–483) μm, 4–6 μm in width, septate; ascus ovoid to broad fusiform, colorless, thick-walled, sessile, 84–116(–121) × 56–79 μm, length/width ratio 1.3–1.6, terminal oculus obvious, large, 17–27 μm diam., 8-spored; ascospores ellipsoid–ovoid, colorless, 20–26 × 10–15 μm, length/width ratio 1.5–2.2.

##### Additional specimens examined.

• A total of 33 specimens were examined, including 27 specimens with voucher numbers HMJAU-PM92759 to HMJAU-PM92785 and six specimens from Herbarium Mycologicum Academiae Sinicae, HMAS 01630, HMAS 14132, HMAS 39017, HMAS 39018, HMAS 39019, and HMAS 39020. Detailed specimen information is presented in Suppl. material 1.

##### Host range and distribution.

On *Rosa* (davurica, *koreana*, multiflora, rugosa, sp.), *Rosaceae*; Asia (China, Korea).

##### Notes.

This species was once proposed as *Sphaerotheca rosae* by Zhao (1981) and accepted by Zheng and Yu (1987), whereas most other authors considered this name a synonym of *P. pannosa* (Braun 1987, 1995; Shin 2000; Liu 2010; Braun and Cook 2012). After our detailed examinations, including the results of sequence analyses, Zhao’s (1981) taxonomic treatment can be confirmed, i.e., *S. rosae* can be confirmed as a species. *P. rosae* is different from *P. pannosa* in (1) forming chasmothecia mostly on the lower surfaces of the leaves; (2) having longer conidiophores and foot cells; (3) smaller chasmothecia and asci with larger terminal oculi; and (4) longer chasmothecial appendages. Sequences of *S. rosae*, including ex-type sequences (PX239400 and PX239279 from HMAS 39018; PX239428 and PX239307 from HMAS 39020), grouped together in a monophyletic, highly supported clade separate from the *P. pannosa* clade. As a result, *S. rosae* should be reinstated as the new combination *Podosphaera rosae*. *P. pannosa* reported by Lee et al. (2011) on *Rosa rugosa* in Korea is morphologically and phylogenetically consistent with *P. rosae*.

#### Podosphaera rosae-xanthinae

Taxon classificationFungiHelotialesErysiphaceae

D.-N. Jin & S.-Y. Liu sp. nov.

7C92D18C-B9D5-56D4-AB37-B080690134EE

861535

Fig. 9

##### Etymology.

Epithet derived from the name of the type host species, *Rosa xanthina*.

**Figure 9.**
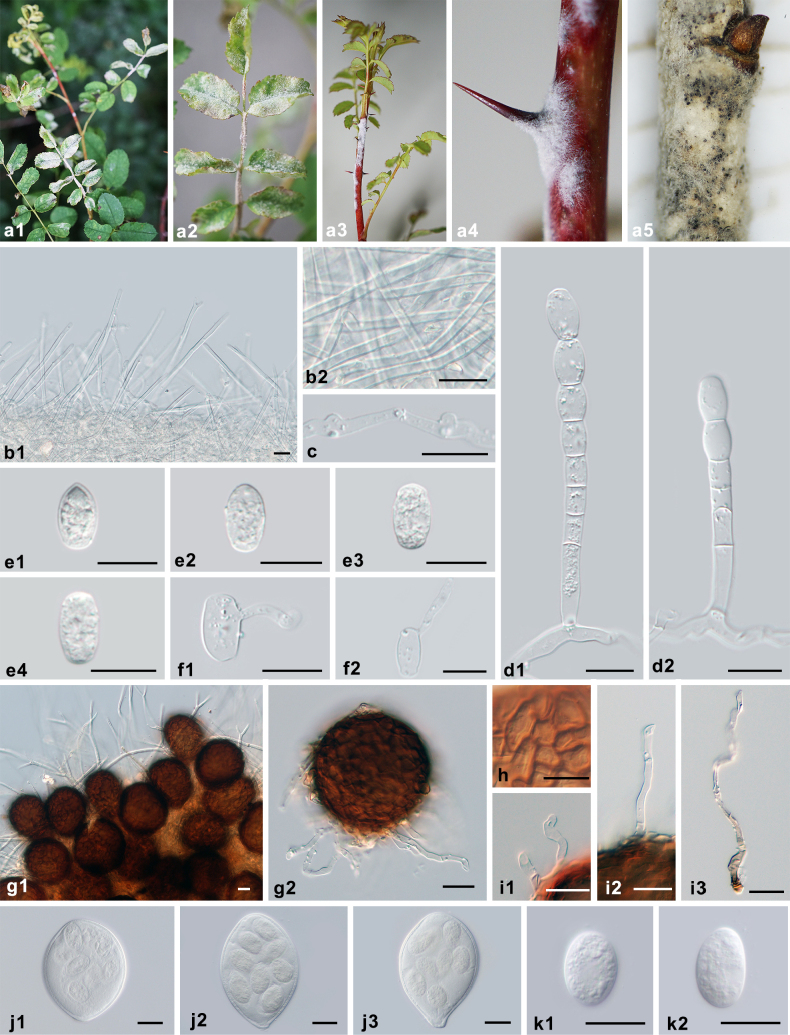
Symptoms and morphology of *Podosphaera rosae-xanthinae* on *Rosa xanthina* (HMJAU-PM92790). **a1–a5** Symptoms. **b1, b2** Secondary hyphae. **c** Hyphal appressoria. **d1, d2** Conidiophores with catenescent conidia. **e1–e4** Conidia. **f1, f2** Germ tubes. **g1, g2** Chasmothecia. **h** Peridium cells. **i1–i3** Appendages. **j1–j3** Asci. **k1, k2** Ascospores. Scale bars: 20 μm (**b1–k2**).

##### Type.

***Holotype***: China • Jilin, Changchun, on *Rosa xanthina*, 8 Aug. 2023, D.N. Jin & X.L. Wu (HMJAU-PM92790). Ex-holotype sequences: PX239433 (ITS), PX239312 (28S), PX239920 (IGS). Isotype: HMAS 354228.

##### Diagnosis.

Close to *Podosphaera pannosa*, but differing in having shorter conidiophores and smaller asci, and forming a separate well-supported species clade in the phylogenetic analysis.

##### Description.

Mycelium on stems and both sides of leaves, more abundant on the lower surface of the leaves, forming irregular patches or effuse, persistent, infected shoots often curled and distorted, primary mycelium white, secondary mycelium in dense, pannose patches on the stems; primary hyphae hyaline, thin-walled, 2.5–5 μm wide, secondary hyphae hyaline, thick-walled, 3.5–5.5 μm wide, sparsely branched; hyphal appressoria indistinct to nipple-shaped, solitary or side by side; conidiophores arising from the upper surface of mother cells, erect, 48–91(–96) × 7–9.5 μm, foot cells straight, cylindrical, (24–)26–56(–61) × 6.5–9.5 μm, followed by 2–3 shorter cells, forming catenescent conidia; primary conidia obovoid, secondary conidia doliiform to ellipsoid, 18.5–25 × 11–14.5 μm, length/width ratio 1.4–1.9; germ tubes almost terminal to almost lateral, short. Chasmothecia gregarious, subglobose to globose, immersed in the secondary mycelial patches, mostly on the stems, (71–)73–96 μm diam.; peridium cells round to irregularly shaped, 5–21(–26) μm diam.; appendages mycelioid, simple, short, arising from the lower half of chasmothecia, usually curved, hyaline throughout or light brown below, paler towards the apex, septate, variable in length, usually 0.2–1 times as long as the chasmothecial diam.; ascus ovoid to ellipsoid, colorless, thick-walled, sessile or short-stalked, 73–102 × 57–71 (–75) μm, length/width ratio 1.2–1.6, terminal oculus inconspicuous to distinct, (11–)14–26 μm, 8-spored; ascospores ellipsoid–ovoid, colorless, 23–28 × 15.5–18.5 μm, length/width ratio 1.4–1.7.

##### Additional specimens examined.

• A total of eight specimens were examined under the vouchers from HMJAU-PM92786 to HMJAU-PM92793. Detailed specimen information is presented in Suppl. material 1.

##### Host range and distribution.

on *Rosa* (xanthina), *Rosaceae*; Asia (China).

##### Notes.

Asexual powdery mildew morphs are common on *Rosa xanthina*. The conidiophores and foot cells are usually shorter than those in *Podosphaera pannosa*. Conidia are also smaller, and the length/width ratio is always less than 2. Chasmothecia are usually formed and are immersed in secondary mycelial patches on twigs in late autumn or early winter. The teleomorph of powdery mildew on *Rosa xanthina* is similar to *P. pannosa*, but differs in having broader asci, sometimes with stalks. Sequences of the powdery mildew on *R. xanthina* form a well-supported, separate species clade in phylogenetic analyses. As a result, a new species, *P. rosae-xanthinae*, is described, which is so far known only on *R. xanthina*.

### Host preference

**Figure 10.**
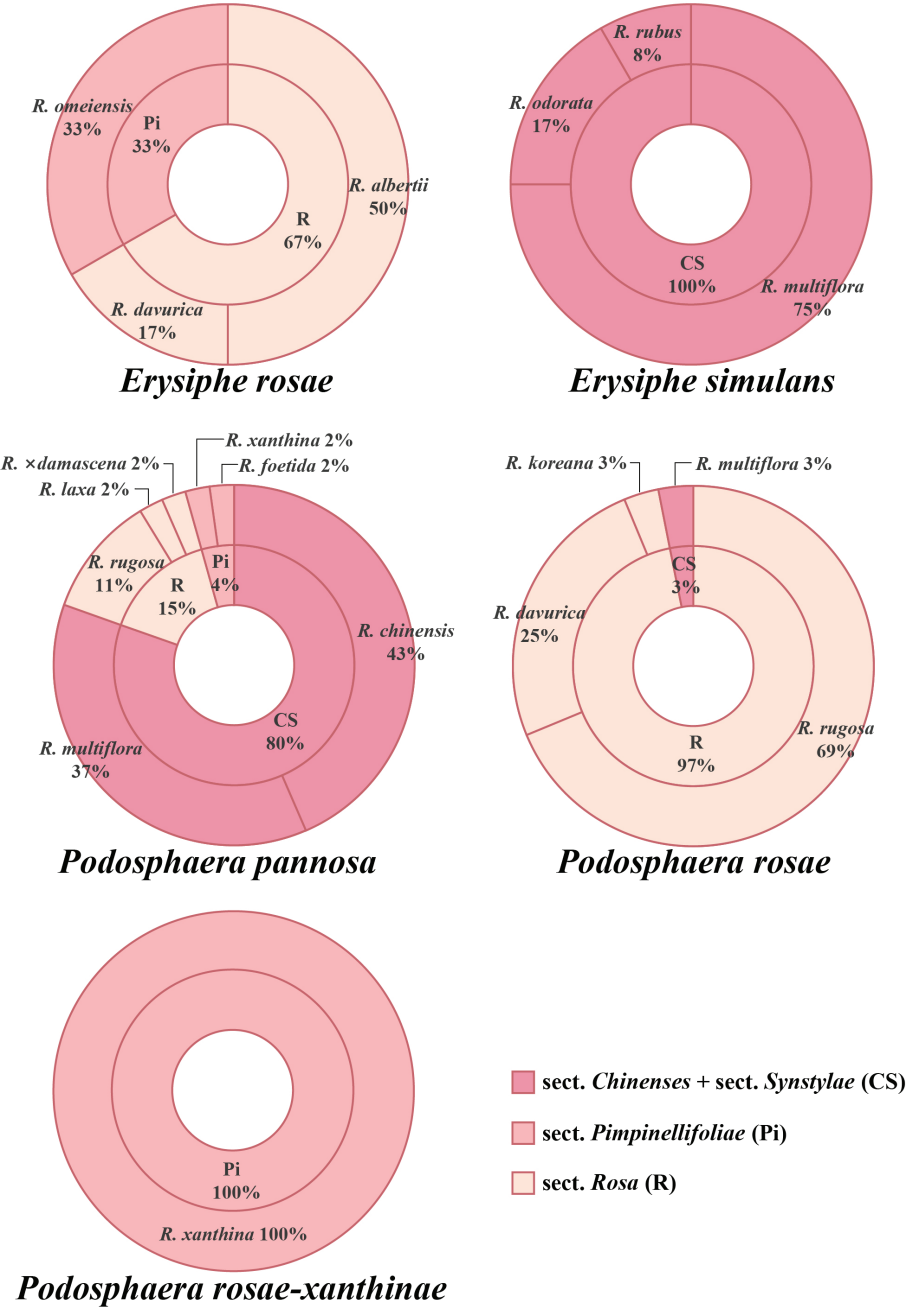
Host frequency of each rose powdery mildew species on *Rosa*. CS = sect. *Chinenses* + sect. *Synstylae*, Pi = sect. *Pimpinellifoliae*, R = sect. *Rosa*.

A total of 104 Chinese specimens with unequivocal host identifications were used for relative frequency (RF) calculations. Host frequency of powdery mildew is shown in Fig. 10. Species within the same powdery mildew genus display host ranges that are both partially specialized and mutually complementary. Among *Erysiphe* species, *E. simulans* is commonly associated with sect. *Chinenses* + sect. *Synstylae* (with *R. multiflora* as the primary host), while *E. rosae* is more prevalent on hosts of sect. *Pimpinellifoliae* and sect. *Rosa* (with *R. omeiensis* and *R. albertii* as the primary hosts). In *Podosphaera*, *P. pannosa* has the broadest host range, infecting seven *Rosa* species, with primary hosts belonging to sect. *Chinenses* + sect. *Synstylae* (with *R. chinensis* and *R. multiflora* as the primary hosts). *P. rosae*, with the second broadest host range, mainly infects hosts within sect. *Rosa* (with *R. rugosa* and *R. davurica* as the primary hosts). Another *Podosphaera* species, *P. rosae-xanthinae*, is so far known only on *R. xanthina*, a species belonging to sect. *Pimpinellifoliae*.

### Distribution patterns of powdery mildews on *Rosa* spp. in China

**Figure 11.**
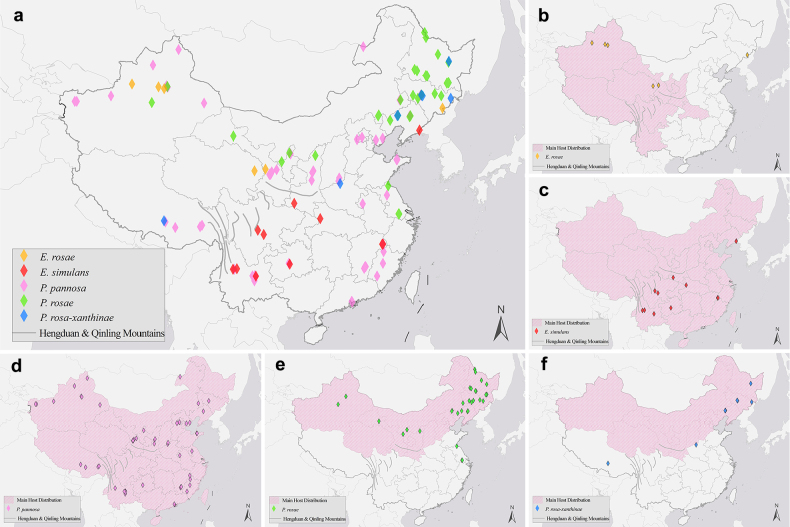
Distribution patterns of rose powdery mildews and the co-distribution with their primary hosts in China. **a** Distribution of five rose powdery mildews in China. **b–f** Co-distribution of rose powdery mildews with their primary hosts: **b***Erysiphe rosae*, **c***E. simulans*, **d***Podosphaera pannosa*, **e***P. rosae*, **f***P. rosae-xanthinae* (distribution ranges of primary hosts are colored in pink on the maps).

The five rose powdery mildew species identified in this study are distributed across 23 provinces in China, and their overall distribution is summarized in Fig. 11a (*Erysiphe rosae*, *E. simulans*, *Podosphaera pannosa*, *P. rosae*, and *P. rosae-xanthinae* are represented by yellow, red, pink, green, and blue points, respectively). The distribution patterns of each species are shown separately in Fig. 11b–f. Additionally, to investigate whether co-distribution exists between each powdery mildew species and its *Rosa* hosts, the main distribution ranges (including both wild and cultivated) of their primary hosts (according to the results of the Host preference part), based on the *Rosa* monograph of Luo et al. (2024), are marked in pink for each powdery mildew species on the maps (Fig. 11b–f).

The results show that powdery mildew on *Rosa* occurs almost nationwide, but different species exhibit distinct distribution patterns (Fig. 11). *Erysiphe rosae* is distributed in the Xinjiang Autonomous Region, Qinghai Province, and Jilin Province. The distribution of *E. simulans* is mainly concentrated in southwestern China and is surrounded by the Hengduan Mountains and Qinling Mountains. *Podosphaera pannosa* is extensively spread across the country, likely due to its wide host range. *P. rosae* is mainly concentrated in northern China. *P. rosae-xanthinae* is mainly found in northeast China (Fig. 11f).

Co-distribution analysis, based on primary *Rosa* hosts, indicates a host-driven distribution of these fungi (Fig. 11b–f). The distributions of *P. pannosa* and *P. rosae* are congruent with the ranges of their primary hosts (Fig. 11d, e). Because of the small sample size of *E. rosae* and *P. rosae-xanthinae*, meaningful predictions regarding co-distribution phenomena are not yet possible (Fig. 11b, f). However, since almost all sampling points fell within the distribution range of the main hosts, it can be assumed that these species follow the same co-distribution trend. *E. simulans* has a narrower distribution range than its primary host (Fig. 11c). It is speculated that, in addition to following co-distribution with its host, its distribution pattern is also influenced by geographical barriers, such as the Hengduan and Qinling Mountains.

## Discussion

### Species diversity of rose powdery mildews in China

Resolving longstanding taxonomic issues clarified and expanded the recognized species diversity of rose powdery mildews. Five species across two genera were identified on *Rosa* in China. Regarding the genus *Erysiphe*, sequences from *E. rosae* provided the first confirmation of this species and the first phylogenetic confirmation that *Medusosphaera* is a synonym of the genus *Erysiphe*. Concurrently, this study confirms *E. simulans* as a single, well-defined species under which the varieties can be removed due to the lack of stable morphological and genetic differentiation. Within the genus *Podosphaera*, we established a taxonomic benchmark for *P. pannosa* s. str. through neotypification and reinstated the independent species status of *P. rosae*, long treated as a synonym of *P. pannosa*. Moreover, the new species *P. rosae-xanthinae* sp. nov. is described. These taxonomic results validate the foresight of early mycologists who recognized the species now known as *P. rosae* (Salmon 1900; Jaczewski 1927; Blumer 1933, 1967; Zheng and Yu 1987), as well as later authors who refined genus- and species-level concepts (Braun and Takamatsu 2000; Bradshaw et al. 2022), while also correcting more than a century of taxonomic confusion largely driven by an overreliance on host identity as a basic diagnostic criterion. More importantly, this discovery clearly indicates that across the vast distribution area of *Rosa* species in China, the diversity of powdery mildew pathogens cannot be represented by a single species. Therefore, future research and management of rose powdery mildew must extend beyond *P. pannosa*. When investigating pathogenic mechanisms, screening for disease resistance genes, developing control strategies, and assessing pathogen population dynamics, it is essential to consider the diverse range of pathogenic species, including *P. rosae*, *P. rosae-xanthinae*, *E. simulans*, and *E. rosae*.

Although *Podosphaera rosae* and *P. rosae-xanthinae* have been segregated from the broadly defined *P. pannosa* species complex, several subclades persist within the *P. pannosa* clade in the ITS+28S+IGS phylogenetic tree (Fig. 3). This suggests the possible existence of distinct formae or even cryptic species within this taxon. However, relying solely on rDNA sequences is insufficient to explore these questions in depth. While multi-gene sequence analyses are increasingly employed in the taxonomy of powdery mildews and have successfully resolved various taxonomic challenges (Qiu et al. 2020; Bradshaw et al. 2022, 2023, 2025b, 2025c; Zhang et al. 2025), such studies remain limited for the genus *Podosphaera* (Bradshaw et al. 2025a). Therefore, future multi-gene phylogenetic analyses based on global samples of powdery mildews on *Rosa* are necessary to clarify this issue.

### Evolutionary patterns of rose powdery mildews

This study reveals that the species diversity, host preferences, and geographical distribution of rose powdery mildews are closely correlated with the phylogenetic lineages of their host plants, indicating a co-evolutionary history with their *Rosa* hosts. Each powdery mildew species exhibited a distinct primary host group, and these host associations were non-random and correlated with the phylogeny of *Rosa*. For example, within *Podosphaera*, *P. pannosa*, *P. rosae*, and *P. rosae-xanthinae* primarily infect different *Rosa* sections (*Chinenses* + *Synstylae*, *Rosa*, and *Pimpinellifoliae*, respectively). This situation also occurs within *Erysiphe*. This species–host section correspondence strongly suggests that co-evolution with *Rosa* hosts has been a primary driver of speciation. These findings are consistent with co-evolutionary patterns documented in other powdery mildew and host systems (Matsuda and Takamatsu 2003; Takamatsu et al. 2010; Guan et al. 2022). Species appear to achieve niche differentiation through specialization on distinct host lineages, thereby reducing interspecific competition. Species within the genus *Erysiphe* exhibit intergeneric niche overlap with *Podosphaera* species on some hosts (e.g., sect. *Synstylae*), indicating that pathogens from different genera may employ different molecular mechanisms to infect the same host group. Alternatively, these pathogens may have co-evolved on a different host genus or species and then jumped to these other hosts.

The distribution analysis further reinforces the co-evolutionary pattern, revealing a host-driven distribution. The distribution of *P. pannosa* and *P. rosae* closely matches the ranges of their respective primary host groups. An interesting exception is *E. simulans*, whose current distribution is much smaller than the modern distribution range of its primary host in sect. *Synstylae*. This suggests that the origin of *E. simulans* may coincide with the center of origin for sect. *Synstylae* (Cheng et al. 2025), but its subsequent dispersal failed to fully keep pace with the radiative evolution of its hosts, potentially limited by geographical barriers such as the Qinling and Hengduan Mountains. However, the distribution of *E. simulans* in Hubei and Liaoning Provinces indicates that the fungus may be influenced by the expansion of its hosts, gradually expanding its distribution to the east. As a major center of origin and diversity for *Rosa*, China contains substantial species richness (Luo et al. 2024). The highly diverse powdery mildew taxa revealed in this study, encompassing almost all currently known powdery mildew species infecting *Rosa* globally (with the exception of *E. karisiana*, which is known only from its type specimen), further support the notion that host diversity is a key driver of powdery mildew diversity.

However, co-evolution with *Rosa* is likely not the sole evolutionary force shaping the history of these pathogens. Although diversification appears to track host radiation within *Rosa*, host jumps at the genus and family levels must also be considered for powdery mildews. In addition to the *Rosa* hosts, *Podosphaera pannosa* infects plants from six other genera in five families. These non-*Rosa* hosts exhibit a phylogenetically discontinuous distribution, a pattern that strongly implies repeated host jumps in their evolutionary history. Notably, the *Rosa* hosts of *P. pannosa* are predominantly commercially cultivated ornamental species and cut-flower cultivars that are extensively traded worldwide. As Thines et al. (2023) emphasized, host jumps and geographic expansions have been dramatically accelerated in the Anthropocene by global trade. Therefore, the exceptionally broad host range of *P. pannosa* likely represents a recent, human-mediated host range expansion. Taken together, the evolutionary history of rose powdery mildews likely exhibits a bimodal pattern. Host–pathogen correspondence at lower taxonomic levels primarily reflects ancient co-speciation, whereas broad and phylogenetically discontinuous host ranges reflect recent, globalization-driven host jumps.

## Conclusion

We successfully resolved longstanding taxonomic confusions, revealing that *Rosa* species host at least five different powdery mildew species across the genera *Erysiphe* (*E. rosae* s. lat. and *E. simulans*) and *Podosphaera* (*P. pannosa* s. str., *P. rosae* comb. nov., and *P. rosae-xanthinae* sp. nov.) in China. Host preferences and distribution patterns of these fungi indicate that the long-term co-evolutionary relationship between *Rosa* and their powdery mildews serves as the core driver of pathogen speciation and niche specialization, highlighting the importance of *Rosa* hosts to the evolutionary history of powdery mildew. Future disease-resistance breeding efforts must account for pathogen diversity by targeting the locally dominant powdery mildew species and tailoring resistant cultivars to specific ecological regions and *Rosa* species. Furthermore, multi-gene and genomic analyses are needed to more deeply resolve genetic and population-level diversity and to elucidate the co-evolutionary mechanisms underlying host–pathogen interactions.

## Supplementary Material

XML Treatment for Erysiphe rosae

XML Treatment for Erysiphe simulans

XML Treatment for Podosphaera pannosa

XML Treatment for Podosphaera rosae

XML Treatment for Podosphaera rosae-xanthinae

## Additional information

### Use of AI

No use of AI was reported.

### Adherence to national and international regulations

All the fungal strains used in this study have been legally obtained, respecting the Convention on Biological Diversity (Rio Convention).

### Author ORCIDs

Dan-Ni Jin https://orcid.org/0000-0003-2739-3740

Michael Bradshaw https://orcid.org/0000-0002-7133-4679

Shu-Yan Liu https://orcid.org/0000-0001-9082-1053

### Data availability

Sequences obtained in this study have been deposited in GenBank, with all additional data included in the manuscript and its supplements.

## Supplementary materials

Supplementary material 1 Supplementary data 1 Data type docx Explanation note Information of specimens examined in this study. https://binary.pensoft.net/file/1553871This dataset is made available under the Open Database License (http://opendatacommons.org/licenses/odbl/1.0/). The Open Database License (ODbL) is a license agreement intended to allow users to freely share, modify, and use this Dataset while maintaining this same freedom for others, provided that the original source and author(s) are credited.Author: Dan-Ni Jin

Supplementary material 2 Supplementary data 2 Data type fas Explanation note Alignment for Fig. 1. https://binary.pensoft.net/file/1553872This dataset is made available under the Open Database License (http://opendatacommons.org/licenses/odbl/1.0/). The Open Database License (ODbL) is a license agreement intended to allow users to freely share, modify, and use this Dataset while maintaining this same freedom for others, provided that the original source and author(s) are credited.Author: Dan-Ni Jin

Supplementary material 3 Supplementary data 3 Data type fas Explanation note Alignment for Fig. 2. https://binary.pensoft.net/file/1553873This dataset is made available under the Open Database License (http://opendatacommons.org/licenses/odbl/1.0/). The Open Database License (ODbL) is a license agreement intended to allow users to freely share, modify, and use this Dataset while maintaining this same freedom for others, provided that the original source and author(s) are credited.Author: Dan-Ni Jin

Supplementary material 4 Supplementary data 4 Data type fas Explanation note Alignment for Fig. 3. https://binary.pensoft.net/file/1553874This dataset is made available under the Open Database License (http://opendatacommons.org/licenses/odbl/1.0/). The Open Database License (ODbL) is a license agreement intended to allow users to freely share, modify, and use this Dataset while maintaining this same freedom for others, provided that the original source and author(s) are credited.Author: Dan-Ni Jin

## References

[B1] Blumer S (1933) Die Erysiphaceen Mitteleuropas unter besonderer Berücksichtigung der Schweiz. Beiträge zur Kryptogamenflora der Schweiz 7. Druck und Verlag von Gebr. Fretz A. G., Zurich, Switzerland, 483 pp.

[B2] Blumer S (1967) Echte Mehltaupilze (*Erysiphaceae*) Ein Bestimmungsbuch für die in Europa Vorkommenden Arten. Gustav Fischer Verlag, Jena, Germany, 436 pp.

[B3] Božanić Tanjga B, Ljubojević M, Ðukić A et al. (2022) Selection of garden roses to improve the ecosystem services they provide. Horticulturae 8: 883. 10.3390/horticulturae8100883

[B4] Bradshaw M, Boufford D, Braun U et al. (2024) An in-depth evaluation of powdery mildew hosts reveals one of the world’s most common and widespread groups of fungal plant pathogens. Plant Disease 108: 576–581. 10.1094/PDIS-07-23-1471-RE 37755416

[B5] Bradshaw M, Braun U, Crouch U et al. (2025a) Phylogeny and taxonomy of the genera of *Erysiphaceae*, part 8: *Podosphaera* sect. *Tridactyla*. Mycologia 118: 116–129. 10.1080/00275514.2025.2554558 41191582

[B6] Bradshaw M, Braun U, Khodaparast SA et al. (2025b) Phylogeny and taxonomy of the genera of *Erysiphaceae*, part 7: *Phyllactinieae*. Mycologia 117: 640–700. 10.1080/00275514.2025.2476375 40258175

[B7] Bradshaw M, Braun U, Mitchell JK et al. (2025c) Phylogeny and taxonomy of the genera of *Erysiphaceae*, part 6: *Erysiphe* (the “*Microsphaera* lineage” part 2). Mycologia 117: 110–165. 10.1080/00275514.2024.2386230 39495585

[B8] Bradshaw M, Braun U, Pfister DH (2022) Phylogeny and taxonomy of the genera of *Erysiphaceae*, part 1: *Golovinomyces*. Mycologia 114: 964–993. 10.1080/00275514.2022.2115419 36223598

[B9] Bradshaw M, Braun U, Pfister DH (2023) Phylogeny and taxonomy of the genera of *Erysiphaceae*, part 4: *Erysiphe* (the “*Uncinula* lineage”). Mycologia 115: 871–903. 10.1080/00275514.2023.2230853 37676759

[B10] Bradshaw M, Tobin PC (2020) Sequencing herbarium specimens of a common detrimental plant disease (powdery mildew). Phytopathology 110: 1248–1254. 10.1094/PHYTO-04-20-0139-PER 32407253

[B11] Braun U (1987) A monograph of the *Erysiphales* (powdery mildews). Beihefte zur Nova Hedwigia, Weinheim, Germany, 700 pp.

[B12] Braun U (1995) The powdery mildews (*Erysiphales*) of Europe. VEB Gustav Fischer Verlag, Jena, Germany, 337 pp.

[B13] Braun U, Cook RTA (2012) Taxonomic manual of the *Erysiphales* (Powdery Mildews). CBS Biodiversity Series 11. CBS-KNAW Fungal Biodiversity Centre, Utrecht, The Netherlands, 707 pp.

[B14] Braun U, Takamatsu S (2000) Phylogeny of *Erysiphe*, *Microsphaera*, Uncinula (Erysipheae) and *Cystotheca*, *Podosphaera*, Sphaerotheca (Cystotheceae) inferred from rDNA ITS sequences — some taxonomic consequences. Schlechtendalia 4: 1–33. 10.25673/90008

[B15] Carbone I, Kohn LM (1999) A method for designing primer sets for speciation studies in filamentous ascomycetes. Mycologia 91: 553–556. 10.1080/00275514.1999.12061051

[B16] Cheng B-X, Zhao K, Zhou M-C et al. (2025) Phenotypic and genomic signatures across wild *Rosa* species open new horizons for modern rose breeding. Nature Plants 11: 775–789. 10.1038/s41477-025-01955-5 40186008

[B17] Chinese Pharmacopoeia Commission (2020) Pharmacopoeia of the People’s Republic of China 2020 Edition Vol. 1. China Medical Science Press, Beijing, China, 1902 pp.

[B18] Cui W-H, Du X-Y, Zhong M-C et al. (2022) Complex and reticulate origin of edible roses (*Rosa*, *Rosaceae*) in China. Horticulture Research 9: uhab051. 10.1093/hr/uhab051 PMC8788372 35031798

[B19] Cunnington JH, Takamatsu S, Lawrie AC et al. (2003) Molecular identification of anamorphic powdery mildews (*Erysiphales*). Australasian Plant Pathology 32: 421–428. 10.1071/AP03045

[B20] Edler D, Klein J, Antonelli A et al. (2021) raxmlGUI 2.0 beta: a graphical interface and toolkit for phylogenetic analyses using RAxML. Methods in Ecology and Evolution 12: 373–377. 10.1111/2041-210X.13512

[B21] Fayaz F, Singh K, Gairola S et al. (2024) A comprehensive review on phytochemistry and pharmacology of *Rosa* species (*Rosaceae*). Current Topics in Medicinal Chemistry 24: 364–378. 10.2174/0115680266274385231023075011 37937578

[B22] Feng J, Guan G-X, Wu X-L et al. (2025a) Phylogeny and taxonomy of *Acer* powdery mildews, including genera *Sawadaea* and *Takamatsuella* (*Erysiphaceae*, *Ascomycota*). Studies in Mycology 112: 1–38. 10.3114/sim.2025.112.01 PMC12786631 41522874

[B23] Feng J, Liu S-Y, Braun U et al. (2022) Discovery of a cryptic species, *Erysiphe salicina* sp. nov., and reconstruction of the phylogeny of powdery mildews on *Populus* and *Salix* spp. Mycological Progress 21: 54. 10.1007/s11557-022-01793-1

[B24] Feng J, Wang S-B, Liu S-Y et al. (2025b) Taxonomy and phylogeny of *Erysiphe* species on *Liquidambar formosana* (*Hamamelidaceae*). New Zealand Journal of Botany 63: 1567–1580. 10.1080/0028825X.2025.2516573

[B25] Golovin PN, Gamalitskaya NA (1962) Novyi rod iz semeistva *Erysiphaceae*. Botanicheskie Materialy Otdela Sporovyh Rasteniy 15: 91–93.

[B26] Gu C-Z, Robertson KR (2003) *ROSA*. In: Wu CY, Raven PH (Eds) Flora of China Vol 9. Science Press/Missouri Botanical Garden Press, Beijing/St. Louis, 339–381.

[B27] Guan G-X, Liu S-Y, Braun U et al. (2022) A cryptic powdery mildew (*Golovinomyces hieraciorum* sp. nov.) on *Hieracium* and *Pilosella (Compositae)*. Phytopathologia Mediterranea 61: 107–117. 10.36253/phyto-12992

[B28] Hegde AS, Gupta S, Sharma S et al. (2022) Edible rose flowers: A doorway to gastronomic and nutraceutical research. Food Research International 162: 111977. 10.1016/j.foodres.2022.111977 36461291

[B29] Hirata T, Takamatsu S (1996) Nucleotide diversity of rDNA internal transcribed spacers extracted from conidia and cleistothecia of several powdery mildew fungi. Mycoscience 37: 283–288. 10.1007/BF02461299

[B30] Homma Y (1937) *Erysiphaceae* of Japan. Journal of the Faculty of Agriculture, Hokkaido University 38: 183–461. https://hdl.handle.net/2115/12712

[B31] Jaczewski AA (1927) Karmanny opredelitel gribov. Vyp. 2. Muchnisto-rosyanye griby. Mikologicheskaya Laboratoriya Imeni Professora A.A. Jaczewskogo, Gosudarstvennogo Instituta Opytnoy Agronomii, Leningrad, 128 pp.

[B32] Jiang L (2024) Advancements and prospects in China’s edible rose industry: breeding, cultivation, and processing. Environment, Development and Sustainability. Advance online publication. 10.1007/s10668-024-05765-1

[B33] Jin D-N, Kiss L, Takamatsu S et al. (2021) Hidden diversity of powdery mildews belonging to the recently re-discovered genus *Salmonomyces*. Mycological Progress 20: 1009–1018. 10.1007/s11557-021-01679-8

[B34] Junell L (1967) *Erysiphaceae* of Sweden. Vol. 19, Lundequistska bokhandeln, Uppsala, Sweden, 117 pp.

[B35] Kiss L, Vaghefi N, Bransgrove K et al. (2020) Australia: a continent without native powdery mildews? The first comprehensive catalog indicates recent introductions and multiple host range expansion events, and leads to the re-discovery of *Salmonomyces* as a new lineage of the *Erysiphales*. Frontiers in Microbiology 11: 1571. 10.3389/fmicb.2020.01571 PMC7378747 32765452

[B36] Kumar S, Stecher G, Li M et al. (2018) MEGA X: Molecular evolutionary genetics analysis across computing platforms. Molecular Biology and Evolution 35: 1547–1549. 10.1093/molbev/msy096 PMC5967553 29722887

[B37] Lee S-H, Han K-S, Park J-H et al. (2011) Occurrence of *Podosphaera pannosa* teleomorph on *Rosa rugosa* from Korea. The Plant Pathology Journal 27: 398. 10.5423/PPJ.2011.27.4.398

[B38] Li J-H, Guo Z-X, Luo Y et al. (2021) Chitosan can induce *Rosa roxburghii* Tratt. against *Sphaerotheca* sp. and enhance its resistance, hotosynthesis, yield, and quality. Horticulturae 7: 289. 10.3390/horticulturae7090289

[B39] Liu T-Z (2010) The *Erysiphaceae* of Inner Mongolia. Inner Mongolia Science and Technology Press, Chifeng, China, 322 pp.

[B40] Liu L, Hui L-C, Yu S-R et al. (2022) *Erysiphe ruyongzhengiana* sp. nov., a new powdery mildew species on *Aristolochia debilis*, belonging to the *Erysiphe aquilegiae* clade. Mycoscience 63: 169–175. 10.47371/mycosci.2022.05.005 PMC10042307 37090474

[B41] Luo L, Yang Y-Y, Zhang Q-X (2024) Genus *Rosa* L. in China. China Forestry Publishing House, Beijing, China, 552 pp.

[B42] Marmolejo J, Siahaan SAS, Takamatsu S et al. (2018) Three new records of powdery mildews found in Mexico with one genus and one new species proposed. Mycoscience 59: 1–7. 10.1016/j.myc.2017.06.010

[B43] Matsuda S, Takamatsu S (2003) Evolution of host-parasite relationships of *Golovinomyces* (*Ascomycete*: *Erysiphaceae*) inferred from nuclear rDNA sequences. Molecular Phylogenetics and Evolution 27: 314–327. 10.1016/S1055-7903(02)00401-3 12695094

[B44] Mori Y, Sato Y, Takamatsu S (2000) Evolutionary analysis of the powdery mildew fungi using nucleotide sequences of the nuclear ribosomal DNA. Mycologia 92: 74–93. 10.2307/3761452

[B45] Nomura Y (1997) Taxonomical study of *Erysiphaceae* of Japan. Yokendo Ltd, Tokyo, Japan, 281 pp.

[B46] Nylander JAA (2004) MrModeltest V2. Program distributed by the author. Evolutionary Biology Centre, Uppsala University. https://github.com/nylander/MrModeltest2

[B47] Prasad M, Balasubramaniam LM, Priya MDL et al. (2025) Exploring the potential of *Rosa chinensis*, *Rosa cymosa*, and *Rosa indica* in oral disease prevention: A multifaceted approach. Journal of Oral and Maxillofacial Pathology 29: 41–49. 10.4103/jomfp.jomfp_36_25 PMC12002594 40248632

[B48] Qiu P-L, Braun U, Li Y et al. (2019) *Erysiphe deutziicola* sp. nov. (*Erysiphaceae*, *Ascomycota*), a powdery mildew species found on *Deutzia parviflora* (*Hydrangeaceae*) with unusual appendages. MycoKeys 51: 97–106. 10.3897/mycokeys.51.34956 PMC6520331 31139005

[B49] Qiu P-L, Liu S-Y, Bradshaw M et al. (2020) Multi-locus phylogeny and taxonomy of an unresolved, heterogeneous species complex within the genus *Golovinomyces* (*Ascomycota*, *Erysiphales*), including *G. ambrosiae*, *G. circumfusus* and *G. spadiceus*. BMC Microbiology 20: 51. 10.1186/s12866-020-01731-9 PMC7059721 32138640

[B50] Raymond O, Gouzy J, Just J et al. (2018) The *Rosa* genome provides new insights into the domestication of modern roses. Nature Genetics 50: 772–777. 10.1038/s41588-018-0110-3 PMC5984618 29713014

[B51] Ronquist F, Huelsenbeck JP (2003) MrBayes 3: Bayesian phylogenetic inference under mixed models. Bioinformatics 19: 1572–1574. 10.1093/bioinformatics/btg180 12912839

[B52] Salmon ES (1900) A monograph of the *Erysiphaceae*. Memoirs of the Torrey Botanical Club, Vol. 9, 292 pp.

[B53] Scholin CA, Herzog M, Sogin M et al. (1994) Identification of group and strain-specific markers for globally distributed *Alexandrium (Dinophyceae)*. II. sequence analysis of a fragment of the LSU rRNA gene. Journal of Phycology 30: 999–1011. 10.1111/j.0022-3646.1994.00999.x

[B54] Shin HD (2000) *Erysiphaceae* of Korea. National Institute of Agricultural Science and Technology, Suwon, Korea, 320 pp.

[B55] Swofford DL (2002) PAUP*. Phylogenetic analysis using parsimony (and other methods). Version 4.0b10. Sinauer Associates, Sunderland, MA, USA.

[B56] Tang S-Y, Guan G-X, Liu S-Y (2018) Advances on the Taxonomy of *Erysiphales* in China. Journal of Fungal Research 16: 138–149. 10.13341/j.jfr.2018.8006

[B57] Tang S-R, Jiang W-T, Qiu P-L et al. (2017) *Podosphaera paracurvispora* (*Erysiphaceae*, *Ascomycota*), a new powdery mildew species on *Pyrus* from China. Mycoscience 58: 116–120. 10.1016/j.myc.2016.11.004

[B58] Takamatsu S, Hirata T, Sato Y et al. (1999) Phylogenetic relationships of *Microsphaera* and *Erysiphe* section *Erysiphe* (powdery mildews) inferred from the rDNA ITS sequences. Mycoscience 40: 259–268. 10.1007/BF02463963

[B59] Takamatsu S, Niinomi S, Harada M et al. (2010) Molecular phylogenetic analyses reveal a close evolutionary relationship between *Podosphaera* (*Erysiphales*: *Erysiphaceae*) and its rosaceous hosts. Persoonia 24: 38–48. 10.3767/003158510X494596 PMC2890165 20664759

[B60] Thines M, Seebens H, Aime MC et al. (2023) Host switching and geographic expansions in (hemi)biotrophic plant pathogens. In: Pöggeler S, James T (Eds) The Mycota 14: Evolution of Fungi and Fungal-Like Organisms. Springer Nature Switzerland AG, Cham, Switzerland, 123–148. 10.1007/978-3-031-29199-9

[B61] Walsh PS, Metzger DA, Higuchi R (1991) Chelex 100 as a medium for simple extraction of DNA for PCR-Based typing from forensic material. Biotechniques 10: 506–513. 10.2144/000114018 1867860

[B62] Wang L-H, Yang X-M, Tan C-R et al. (2018) Effects of *Bacillus subtilis* (Y1336) on controlling rose powdery mildew and soil nutrient status. Southwest China Journal of Agricultural Sciences 31: 2569–2574. 10.16213/j.cnki.scjas.2018.12.020

[B63] Wang Y-S, Zhao Y-M, Liu X-N et al. (2022) Chemical constituents and pharmacological activities of medicinal plants from *Rosa* genus. Chinese Herbal Medicines 14: 187–209. 10.1016/j.chmed.2022.01.005 PMC9476647 36117670

[B64] White TJ, Bruns T, Lee S et al. (1990) Amplification and direct sequencing of fungal ribosomal RNA genes for phylogenetics. In: Innis MA, Gelfand DH, Sninsky JJ et al. (Eds) PCR Protocols: a guide to methods and applications. Academic Press, San Diego, USA, 315–322.

[B65] Xiang C-Y, Gao F-L, Jakovlić I et al. (2023) Using PhyloSuite for molecular phylogeny and tree‐based analyses. iMeta 2: e87. 10.1002/imt2.87 PMC10989932 38868339

[B66] Yang C-Y, Ma Y-J, Cheng B-X et al. (2020) Molecular evidence for hybrid origin and phenotypic variation of *Rosa* section *Chinenses*. Genes 11: 996. 10.3390/genes11090996 PMC7564265 32854427

[B67] Zhang D, Gao F-L, Jakovlić I et al. (2019) PhyloSuite: An integrated and scalable desktop platform for streamlined molecular sequence data management and evolutionary phylogenetics studies. Molecular Ecology Resources 20: 348–355. 10.1111/1755-0998.13096 31599058

[B68] Zhang T-Y, Gao J-Y, Shen P (2024a) Research on the application of Chinese rose in landscaping. Education Reform and Development 6: 148–154. 10.26689/erd.v6i11.8905

[B69] Zhang Z-Y, Wu X-L, Lv X-X et al. (2025) Discover hidden taxa of *Erysiphe* section *Erysiphe* fungi (*Ascomycota*, *Erysiphaceae*) based on morphology and multilocus phylogeny in China. MycoKeys 118: 119–146. 10.3897/mycokeys.118.154217 PMC12159665 40510759

[B70] Zhang Z, Yang T, Liu Y et al. (2024b) Haplotype-resolved genome assembly and resequencing provide insights into the origin and breeding of modern rose. Nature Plants 10: 1659–1671. 10.1038/s41477-024-01820-x 39394508

[B71] Zhao Z-Y (1981) Taxonomic studies on the genus *Sphaerotheca* of China IV. new species and new combination on *Rosaceae* and *Labiatae*. Acta Microbiologica Sinica 21: 438–442. https://link.cnki.net/doi/10.13343/j.cnki.wsxb.1981.04.007

[B72] Zheng R-Y, Chen G-Q (1979) Taxonomic studies on the genus *Uncinuliella* of China. I. The establishment of the *Uncinuliella* gen. nov. and identification of the Chinese and Japanese species. Acta Microbiologica Sinica 19: 280–291.

[B73] Zheng R-Y, Yu Y-N (1987) Flora Fungorum Sinicorum Vol. 1, *Erysiphales*. Science Press, Beijing, China, 552 pp.

[B74] Zhou L-J, Yu C, Cheng B-X et al. (2020) Volatile compound analysis and aroma evaluation of tea-scented roses in China. Industrial Crops and Products 155: 112735. 10.1016/j.indcrop.2020.112735

[B75] Zhou M-C, Sun Y-L, Luo L et al. (2023) Road to a bite of rosehip: A comprehensive review of bioactive compounds, biological activities, and industrial applications of fruits. Trends in Food Science and Technology 136: 76–91. 10.1016/j.tifs.2023.04.006

[B76] Zuo W-G, Chen Z-B, Xu Y et al. (2014) The study on integrated control techniques of rose powdery mildew. Journal of Kunming University 36: 27–29.

