# Supplementary material for: Revision of powdery mildews (Ascomycota, Erysiphaceae) on Rosa in China: unexpected taxonomic complexity with phytopathological implications
Source: IMA Fungus. 2026 Mar 4;17:e184484. doi: 10.3897/imafungus.17.184484 (PMC12980140; doi:10.3897/imafungus.17.184484)
Supplement: Supplementary material 1 — Supplementary data 1 [file imafungus-17-e184484-s001.docx]

**Table S1.** Information of specimens examined in this study.

| Species | Host | Voucher | Collection Date | Collection Locality | Longitude (°E) | Latitude (°N) | GenBank accession number | | | Note |
| --- | --- | --- | --- | --- | --- | --- | --- | --- | --- | --- |
|  |  |  |  |  |  |  | ITS | 28S | IGS |  |
| *Erysiphe rosae* | *Rosa albertii* | HMAS 36517 | 1973.08.22 | China, Xinjiang | 82.71 | 43.07 | —— | —— | —— |  |
|  | *R. albertii* | HMAS 36518 | 1959.09 | China, Xinjiang | 86.50 | 43.50 | —— | —— | —— |  |
|  | *R. albertii* | HMAS 242383 | 2009.08.03 | China, Xinjiang | 87.50 | 43.84 | PX506005 | PX506007 | PX570626 |  |
|  | *R. davurica* | HMAS 44287 | 1982.09.10 | China, Jilin | 128.07 | 42.05 | —— | —— | —— |  |
|  | *R. omeiensis* | HMJAU-PM92697 | 2024.09.12 | China, Qinghai | 101.65 | 36.31 | PX239338 | PX239218 | PX239837 |  |
|  | *R. omeiensis* | HMAS 138804 | 2003.10.07 | China, Gansu | 102.67 | 36.88 | PX589419 | PX506008 | PX570627 |  |
| *E. simulans* | *R. multiflora* | HMJAU-PM92698 | 2018.11.22 | China, Sichuan | 103.45 | 29.57 | PX239339 | PX239219 | PX239838 |  |
|  | *R. multiflora* | HMJAU-PM92699 | 2019.07.04 | China, Sichuan | 102.68 | 29.98 | PX239340 | PX239220 | PX239839 |  |
|  | *R. multiflora* | HMJAU-PM92700 | 2019.11.13 | China, Yunnan | 100.12 | 25.60 | PX239341 | PX239221 | PX239840 |  |
|  | *R. multiflora* | HMJAU-PM92701 | 2020.09.27 | China, Liaoning | 124.39 | 40.14 | PX239342 | PX239222 | PX239841 |  |
|  | *R. multiflora* | HMJAU-PM92702 | 2020.09.27 | China, Liaoning | 124.35 | 40.10 | PX239343 | PX239223 | PX239842 |  |
|  | *R. multiflora* | HMJAU-PM92703 | 2021.03.25 | China, Jiangxi | 117.89 | 28.45 | PX239344 | PX239224 | —— |  |
|  | *R. multiflora* | HMJAU-PM92704 | 2021.03.25 | China, Jiangxi | 117.98 | 28.45 | PX239345 | PX239225 | PX239843 |  |
|  | *R. multiflora* | HMJAU-PM92705 | 2021.03.30 | China, Yunnan | 102.86 | 25.09 | PX239346 | PX239226 | PX239844 |  |
|  | *R. multiflora* | HMJAU-PM92706 | 2021.04.02 | China, Yunnan | 100.57 | 25.70 | PX239347 | PX239227 | PX239845 |  |
|  | *R. odorata* | HMJAU-PM92707 | 2022.08.15 | China, Hubei | 110.39 | 31.47 | PX239348 | PX239228 | —— |  |
|  | *R. odorata* | HMJAU-PM92708 | 2023.10.24 | China, Shaanxi | 107.07 | 33.07 | PX239349 | PX239229 | —— |  |
|  | *R. multiflora* | HMAS 13631 | 1908 | Japan | —— | —— | —— | —— | —— | Reference specimen of *Uncinuliella simulans* var. *simulans* |
|  | *R. rubus* | HMAS 11418 | 1931.06.17 | China, Guizhou | 106.75 | 26.55 | —— | —— | —— | Holotype of *Uncinuliella simulans* var. *rosae-rubi* |
| *Podosphaera pannosa* | *R. chinensis* | HMJAU-PM92709 | 2014.09.22 | China, Xinjiang | 84.89 | 45.59 | PX239350 | PX239230 | PX239846 |  |
|  | *R. chinensis* | HMJAU-PM92710 | 2018.05.28 | China, Beijing | 116.27 | 40.02 | PX239351 | PX239231 | PX239847 |  |
|  | *R. chinensis* | HMJAU-PM92711 | 2018.09.20 | China, Gansu | 103.65 | 36.05 | PX239352 | PX239232 | PX239848 |  |
|  | *R. chinensis* | HMJAU-PM92712 | 2018.09.20 | China, Gansu | 103.95 | 36.35 | PX239353 | PX239233 | PX239849 |  |
|  | *R. chinensis* | HMJAU-PM92713 | 2018.09.27 | China, Shaanxi | 109.36 | 36.42 | PX239354 | PX239234 | PX239850 |  |
|  | *R. chinensis* | HMJAU-PM92714 | 2018.10.19 | China, Beijing | 115.57 | 39.84 | PX239355 | PX239235 | PX239851 |  |
|  | *R. chinensis* | HMJAU-PM92715 | 2018.10.21 | China, Hebei | 118.18 | 39.63 | PX239356 | PX239236 | PX239852 |  |
|  | *R. chinensis* | HMJAU-PM92716 | 2019.06.26 | China, Yunnan | 102.74 | 25.14 | PX239357 | PX239237 | PX239853 |  |
|  | *R. chinensis* | HMJAU-PM92717 | 2019.06.28 | China, Yunnan | 102.73 | 24.58 | PX239358 | PX239238 | PX239854 |  |
|  | *R. chinensis* | HMJAU-PM92718 | 2019.10.22 | China, Anhui | 115.82 | 32.91 | PX239359 | PX239239 | PX239855 |  |
|  | *R. chinensis* | HMJAU-PM92719 | 2019.11.12 | China, Yunnan | 102.70 | 25.06 | PX239360 | PX239240 | PX239856 |  |
|  | *R. chinensis* | HMJAU-PM92720 | 2021.03.31 | China, Guangdong | 113.97 | 22.52 | PX239361 | PX239241 | PX239857 |  |
|  | *R. chinensis* | HMJAU-PM92721 | 2021.04.01 | China, Guangdong | 113.91 | 22.59 | PX239362 | PX239242 | PX239858 |  |
|  | *R. chinensis* | HMJAU-PM92722 | 2021.08.28 | China, Inner Mongolia | 117.42 | 49.60 | PX239363 | PX239243 | PX239859 |  |
|  | *R. chinensis* | HMJAU-PM92723 | 2023.10.16 | China, Gansu | 103.81 | 36.07 | PX239364 | PX239244 | PX239860 |  |
|  | *R. chinensis* | HMJAU-PM92724 | 2023.10.16 | China, Xinjiang | 76.00 | 39.45 | PX239365 | PX239245 | PX239861 |  |
|  | *R. chinensis* | HMJAU-PM92725 | 2023.10.19 | China, Shaanxi | 109.48 | 36.60 | PX239366 | PX239246 | PX239862 |  |
|  | *R. chinensis* | HMJAU-PM92726 | 2023.10.26 | China, Ningxia | 106.36 | 38.38 | PX239367 | PX239247 | PX239863 |  |
|  | *R. chinensis* | HMJAU-PM92727 | 2023.10.30 | China, Jilin | 125.31 | 43.81 | PX239368 | PX239248 | PX239864 |  |
|  | *R. chinensis* | HMJAU-PM92728 | 2024.09.20 | China, Xizang | 92.59 | 29.14 | PX239369 | PX239249 | PX239865 |  |
|  | *R. damascena* | HMJAU-PM92729 | 2018.08.17 | China, Shanxi | 112.82 | 35.48 | PX239370 | PX239250 | PX239866 |  |
|  | *R. foetida* | HMJAU-PM92730 | 2023.10.14 | China, Xinjiang | 87.60 | 43.80 | PX239371 | PX239251 | PX239867 |  |
|  | *R. multiflora* | HMJAU-PM92731 | 2018.10.22 | China, Hebei | 119.14 | 39.74 | PX239372 | PX239252 | PX239868 |  |
|  | *R. multiflora* | HMJAU-PM92732 | 2019.12.05 | China, Fujian | 117.78 | 26.40 | PX239373 | PX239253 | PX239869 |  |
|  | *R. multiflora* | HMJAU-PM92733 | 2020.05.01 | China, Jiangxi | 115.53 | 26.00 | PX239374 | PX239254 | PX239870 |  |
|  | *R. multiflora* | HMJAU-PM92734 | 2020.05.02 | China, Jiangxi | 115.62 | 26.15 | PX239375 | PX239255 | PX239871 |  |
|  | *R. multiflora* | HMJAU-PM92735 | 2020.05.20 | China, Jiangxi | 115.73 | 26.19 | PX239376 | PX239256 | PX239872 |  |
|  | *R. multiflora* | HMJAU-PM92736 | 2020.06.28 | China, Shandong | 120.74 | 37.34 | PX239377 | PX239257 | —— |  |
|  | *R. multiflora* | HMJAU-PM92737 | 2020.06.28 | China, Shandong | 120.83 | 37.34 | PX239378 | PX239258 | PX239873 |  |
|  | *R. multiflora* | HMJAU-PM92738 | 2021.03.24 | China, Fujian | 117.97 | 27.64 | PX239379 | PX239259 | PX239874 |  |
|  | *R. multiflora* | HMJAU-PM92739 | 2021.03.25 | China, Jiangxi | 117.93 | 28.46 | PX239380 | PX239260 | —— |  |
|  | *R. multiflora* | HMJAU-PM92740 | 2021.03.26 | China, Fujian | 116.99 | 25.11 | PX239381 | PX239261 | PX239875 |  |
|  | *R. multiflora* | HMJAU-PM92741 | 2021.04.03 | China, Yunnan | 100.27 | 25.61 | PX239382 | PX239262 | PX239876 |  |
|  | *R. multiflora* | HMJAU-PM92742 | 2022.08.15 | China, Guizhou | 106.63 | 26.64 | PX239383 | PX239263 | PX239877 |  |
|  | *R. multiflora* | HMJAU-PM92743 | 2022.08.23 | China, Yunnan | 102.76 | 24.97 | PX239384 | PX239264 | PX239878 |  |
|  | *R. multiflora* | HMJAU-PM92744 | 2023.10.15 | China, Xinjiang | 75.75 | 39.38 | PX239385 | PX239265 | PX239879 |  |
|  | *R. multiflora* | HMJAU-PM92745 | 2023.10.17 | China, Xinjiang | 80.30 | 41.20 | PX239386 | PX239266 | PX239880 |  |
|  | *R. multiflora* | HMJAU-PM92746 | 2023.10.20 | China, Shaanxi | 109.27 | 35.58 | PX239387 | PX239267 | PX239881 |  |
|  | *R. multiflora* | HMJAU-PM92747 | 2019.10.16 | China, Jiangsu | 119.00 | 33.64 | PX239388 | PX239268 | PX239882 |  |
|  | *R. rugosa* | HMJAU-PM92748 | 2013.10.08 | China, Xizang | 91.28 | 29.53 | PX239389 | PX239269 | PX239883 |  |
|  | *R. rugosa* | HMJAU-PM92749 | 2018.09.21 | China, Gansu | 103.73 | 36.10 | PX239390 | PX239270 | PX239884 |  |
|  | *R. rugosa* | HMJAU-PM92750 | 2023.10.12 | China, Xinjiang | 87.82 | 43.69 | PX239391 | PX239271 | PX239885 |  |
|  | *R. rugosa* | HMJAU-PM92751 | 2023.10.17 | China, Gansu | 104.58 | 36.61 | PX239392 | PX239272 | PX239886 |  |
|  | *R. rugosa* | HMJAU-PM92752 | 2023.10.21 | China, Xinjiang | 93.50 | 42.80 | PX239393 | PX239273 | PX239887 |  |
|  | *Rosa* sp. | HMJAU-PM92753 | 2018.08.17 | China, Shanxi | 112.92 | 35.80 | PX239394 | PX239274 | —— |  |
|  | *Rosa* sp. | HMJAU-PM92754 | 2020.09.26 | China, Liaoning | 123.32 | 41.80 | PX239395 | PX239275 | PX239888 |  |
|  | *Rosa* sp. | HMJAU-PM92755 | 2021.03.30 | China, Yunnan | 102.46 | 25.09 | PX239396 | PX239276 | PX239889 |  |
|  | *Rosa* sp. | HMJAU-PM92756 | 2024.09.17 | China, Xizang | 95.58 | 29.66 | PX239397 | PX239277 | PX239890 |  |
|  | *Rosa* sp. | HMJAU-PM92757 | 2024.09.17 | China, Xizang | 95.78 | 29.85 | PX239398 | PX239278 | PX239891 |  |
|  | *R. xanthina* | HMJAU-PM92758 | 2024.09.28 | China, Inner Mongolia | 122.17 | 43.61 | PX239399 | —— | —— |  |
|  | *R. laxa* | HMAS 38723 | 1975.07.27 | China, Xinjiang | 88.15 | 47.83 | —— | —— | —— |  |
|  | *Rosa* sp. | NCSLG24638 | 2018.09.27 | Germany, Lower Saxony | —— | —— | PV581762 | PV581762 | PV584310 | Neotype |
| *P. rosae* | *R. davurica* | HMAS 39017 | 1959.10 | China, Jilin | 124.84 | 43.51 | —— | —— | —— |  |
|  | *R. davurica* | HMAS 39018 | 1974.09.16 | China, Liaoning | 123.26 | 41.69 | PX239400 | PX239279 | —— |  |
|  | *R. davurica* | HMJAU-PM92759 | 2016.07.07 | China, Heilongjiang | 127.49 | 50.25 | PX239401 | PX239280 | PX239892 |  |
|  | *R. davurica* | HMJAU-PM92760 | 2018.10.06 | China, Jilin | 124.20 | 43.91 | PX239402 | PX239281 | PX239893 |  |
|  | *R. davurica* | HMJAU-PM92761 | 2019.09.15 | China, Heilongjiang | 126.67 | 45.70 | PX239403 | PX239282 | PX239894 |  |
|  | *R. davurica* | HMJAU-PM92762 | 2020.10.01 | China, Inner Mongolia | 118.89 | 42.27 | PX239404 | PX239283 | PX239895 |  |
|  | *R. davurica* | HMJAU-PM92763 | 2023.10.18 | China, Shaanxi | 109.75 | 38.25 | PX239405 | PX239284 | PX239896 | Epitype |
|  | *R. davurica* | HMJAU-PM92764 | 2024.09.23 | China, Heilongjiang | 128.91 | 47.74 | PX239406 | PX239285 | PX239897 |  |
|  | *R. koreana* | HMAS 39019 | 1977.09.20 | China, Heilongjiang | 126.50 | 45.78 | —— | —— | —— |  |
|  | *R. multiflora* | HMJAU-PM92765 | 2018.10.25 | China, Liaoning | 123.40 | 41.79 | PX239407 | PX239286 | PX239898 |  |
|  | *R. rugosa* | HMJAU-PM92766 | 2016.07.10 | China, Heilongjiang | 127.55 | 49.70 | PX239408 | PX239287 | —— |  |
|  | *R. rugosa* | HMJAU-PM92767 | 2017.09.14 | China, Heilongjiang | 129.58 | 44.59 | PX239409 | PX239288 | PX239899 |  |
|  | *R. rugosa* | HMJAU-PM92768 | 2018.10.03 | China, Jilin | 128.22 | 43.36 | PX239410 | PX239289 | PX239900 |  |
|  | *R. rugosa* | HMJAU-PM92769 | 2018.10.05 | China, Jilin | 127.34 | 43.72 | PX239411 | PX239290 | PX239901 |  |
|  | *R. rugosa* | HMJAU-PM92770 | 2019.09.13 | China, Heilongjiang | 125.01 | 46.60 | PX239412 | PX239291 | —— |  |
|  | *R. rugosa* | HMJAU-PM92771 | 2019.09.13 | China, Heilongjiang | 129.67 | 44.59 | PX239413 | PX239292 | PX239902 |  |
|  | *R. rugosa* | HMJAU-PM92772 | 2019.09.14 | China, Heilongjiang | 125.35 | 46.41 | PX239414 | PX239293 | PX239903 |  |
|  | *R. rugosa* | HMJAU-PM92773 | 2019.09.14 | China, Heilongjiang | 129.38 | 44.57 | PX239415 | PX239294 | PX239904 |  |
|  | *R. rugosa* | HMJAU-PM92774 | 2020.09.30 | China, Liaoning | 120.44 | 41.57 | PX239416 | PX239295 | PX239905 |  |
|  | *R. rugosa* | HMJAU-PM92775 | 2020.10.01 | China, Inner Mongolia | 118.80 | 42.16 | PX239417 | PX239296 | PX239906 |  |
|  | *R. rugosa* | HMJAU-PM92776 | 2020.10.03 | China, Inner Mongolia | 122.27 | 43.61 | PX239418 | PX239297 | PX239907 |  |
|  | *R. rugosa* | HMJAU-PM92777 | 2022.08.27 | China, Jilin | 125.43 | 43.82 | PX239419 | PX239298 | PX239908 |  |
|  | *R. rugosa* | HMJAU-PM92778 | 2023.10.13 | China, Xinjiang | 87.75 | 43.75 | PX239420 | PX239299 | PX239909 |  |
|  | *R. rugosa* | HMJAU-PM92779 | 2023.10.19 | China, Xinjiang | 86.10 | 41.70 | PX239421 | PX239300 | PX239910 |  |
|  | *R. rugosa* | HMJAU-PM92780 | 2023.10.22 | China, Gansu | 98.30 | 39.77 | PX239422 | PX239301 | PX239911 |  |
|  | *R. rugosa* | HMJAU-PM92781 | 2023.10.25 | China, Ningxia | 105.20 | 37.50 | PX239423 | PX239302 | PX239912 |  |
|  | *R. rugosa* | HMJAU-PM92782 | 2023.10.26 | China, Ningxia | 106.21 | 38.48 | PX239424 | PX239303 | —— |  |
|  | *R. rugosa* | HMJAU-PM92783 | 2024.09.21 | China, Heilongjiang | 130.26 | 46.70 | PX239425 | PX239304 | PX239913 |  |
|  | *R. rugosa* | HMJAU-PM92784 | 2024.09.29 | China, Liaoning | 121.63 | 41.99 | PX239426 | PX239305 | PX239914 |  |
|  | *R. rugosa* | HMJAU-PM92785 | 2019.09.30 | China, Jiangsu | 119.28 | 34.62 | PX239427 | PX239306 | PX239915 |  |
|  | *R. rugosa* | HMAS 01630 | 1932.11.16 | China, Jiangsu | 120.24 | 31.51 | —— | —— | —— |  |
|  | *R. rugosa* | HMAS 14132 | 1932.11.16 | China, Jiangsu | 120.31 | 31.50 | —— | —— | —— |  |
|  | *Rosa* sp. | HMAS 39020 | 1974.09.03 | China, Liaoning | 123.41 | 41.77 | PX239428 | PX239307 | —— |  |
| *P. rosae-xanthinae* | *R. xanthina* | HMJAU-PM92786 | 2018.08.19 | China, Shanxi | 113.01 | 35.20 | PX239429 | PX239308 | PX239916 |  |
|  | *R. xanthina* | HMJAU-PM92787 | 2020.09.25 | China, Jilin | 129.50 | 42.91 | PX239430 | PX239309 | PX239917 |  |
|  | *R. xanthina* | HMJAU-PM92788 | 2020.09.29 | China, Liaoning | 121.64 | 42.00 | PX239431 | PX239310 | PX239918 |  |
|  | *R. xanthina* | HMJAU-PM92789 | 2022.07.22 | China, Jilin | 125.51 | 43.71 | PX239432 | PX239311 | PX239919 |  |
|  | *R. xanthina* | HMJAU-PM92790 | 2023.08.08 | China, Jilin | 125.41 | 43.81 | PX239433 | PX239312 | PX239920 | Holotype |
|  | *R. xanthina* | HMJAU-PM92791 | 2024.09.16 | China, Xizang | 91.08 | 29.62 | PX239434 | PX239313 | PX239921 |  |
|  | *R. xanthina* | HMJAU-PM92792 | 2024.09.21 | China, Heilongjiang | 130.36 | 46.80 | PX239435 | PX239314 | —— |  |
|  | *R. xanthina* | HMJAU-PM92793 | 2024.09.29 | China, Liaoning | 121.53 | 41.99 | PX239436 | PX239315 | PX239922 |  |
